# Supplementary figures and images for: Tomatidine is a senotherapeutic compound that improves cognitive function and reduces cellular senescence in aged mice
Source: EMBO Mol Med. 2026 Apr 1;18(5):1530–50. doi: 10.1038/s44321-026-00400-0 (PMC13179369; doi:10.1038/s44321-026-00400-0)

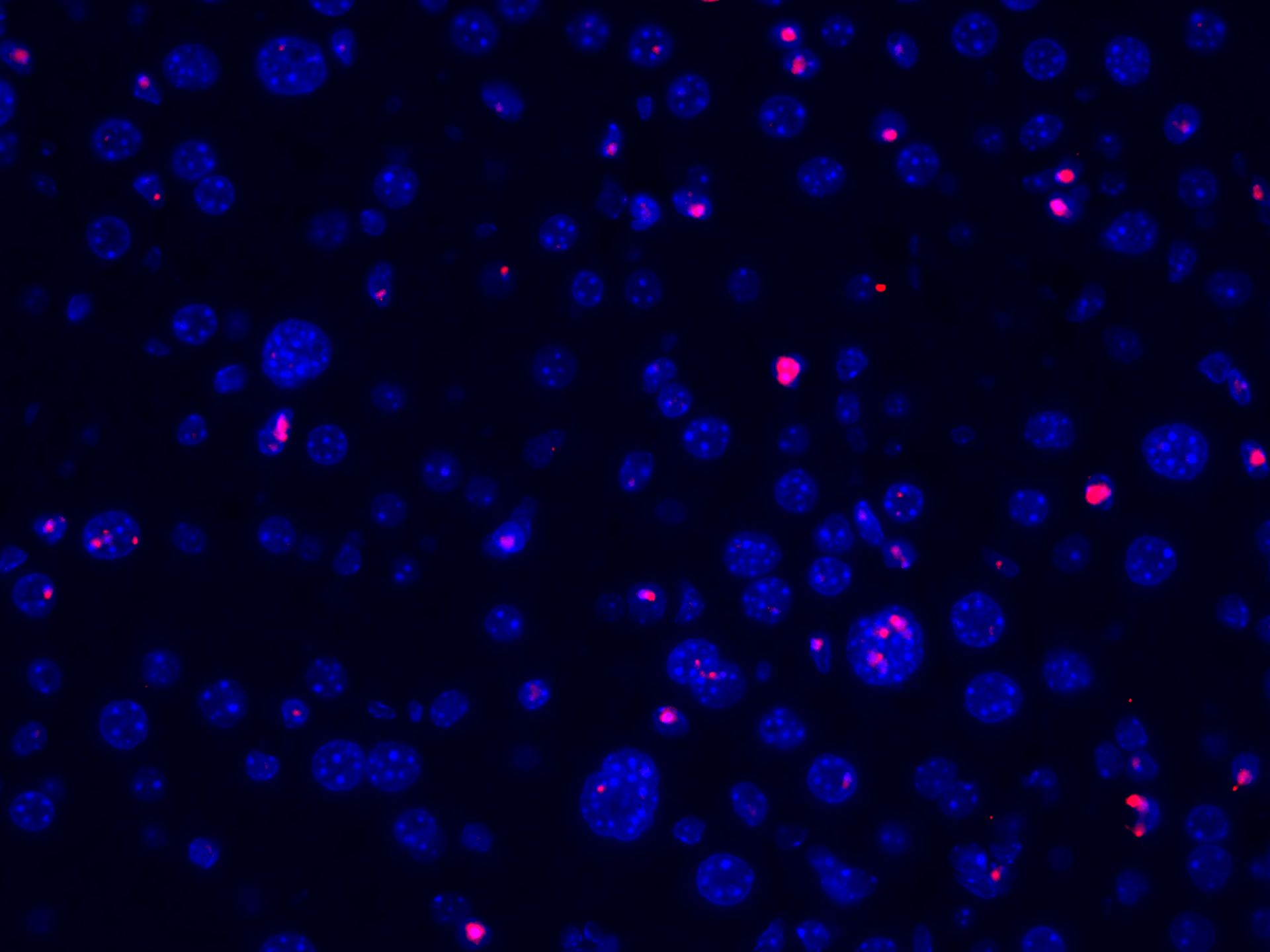

Supplement: Supplementary file 3 — Source data Fig. 2 [file 44321_2026_400_MOESM3_ESM.zip › Fig 2/2a/Liver_DJ879_tomatidine_p21_select image 3.lif - Image 1.png]

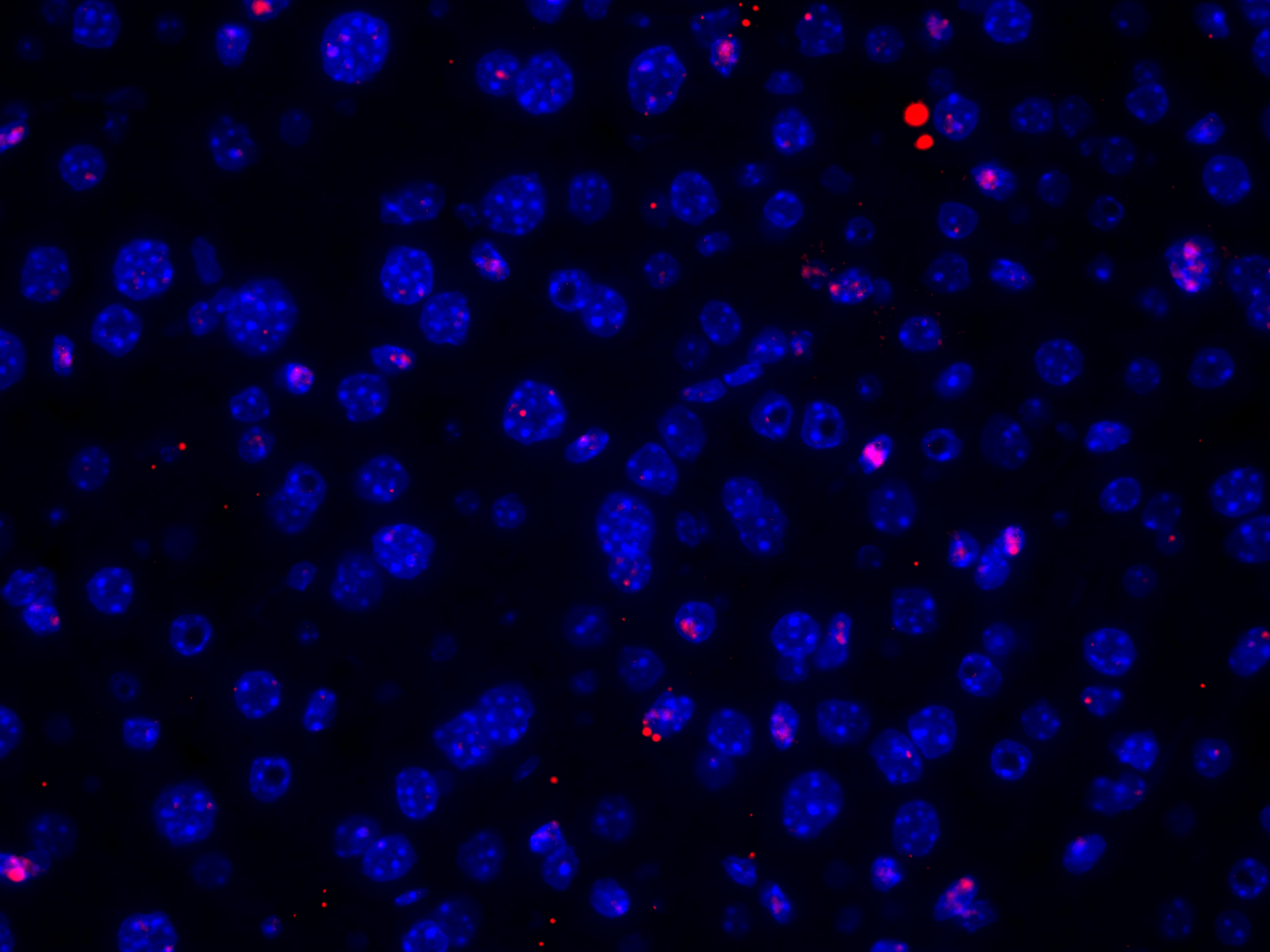

Supplement: Supplementary file 3 — Source data Fig. 2 [file 44321_2026_400_MOESM3_ESM.zip › Fig 2/2a/Liver_DJ891_vehicle_p16_select image 6.lif - Image 1.png]

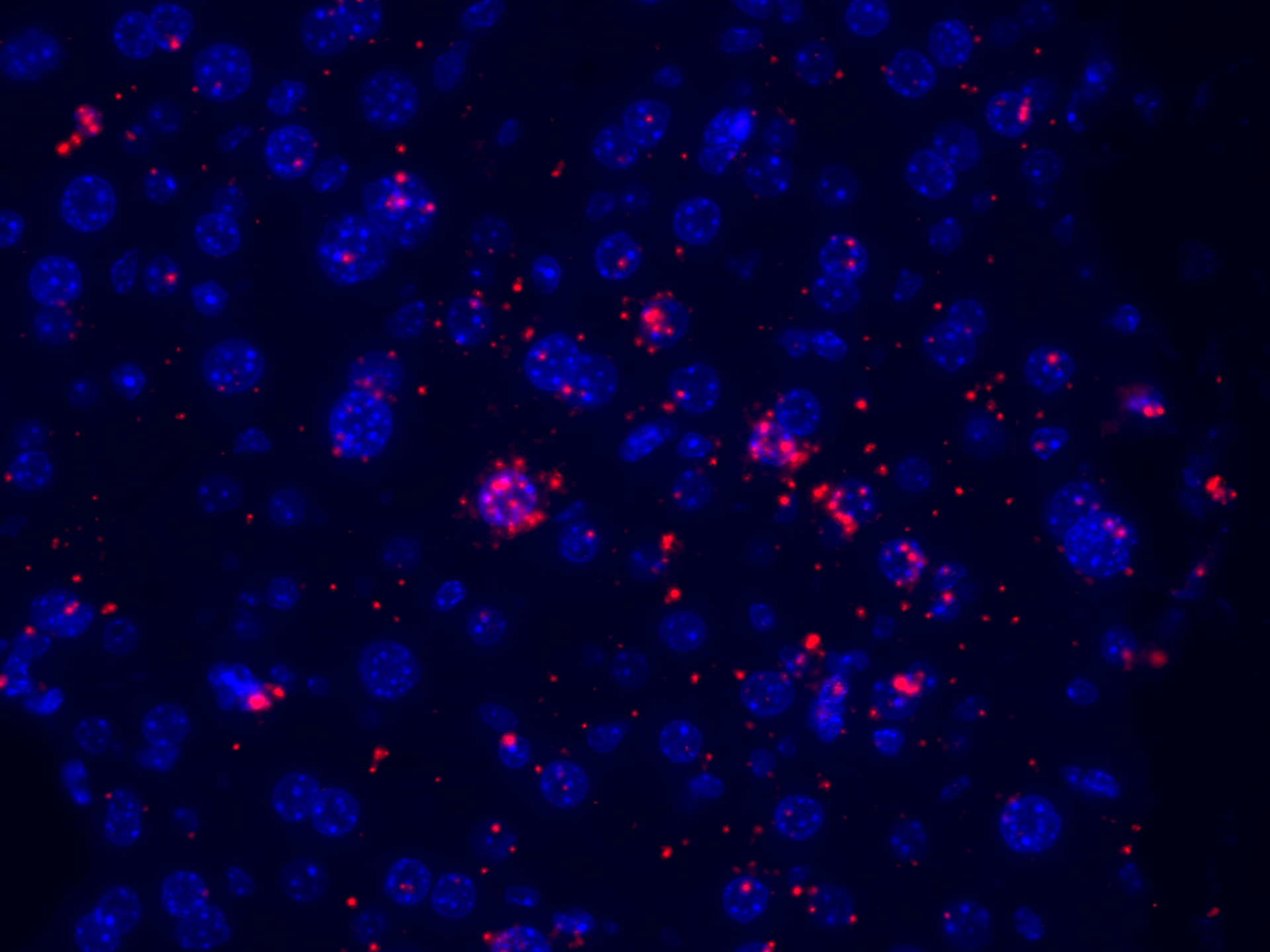

Supplement: Supplementary file 3 — Source data Fig. 2 [file 44321_2026_400_MOESM3_ESM.zip › Fig 2/2a/Liver_DJ891_vehicle_p21_select image 2.lif - Image 7.png]

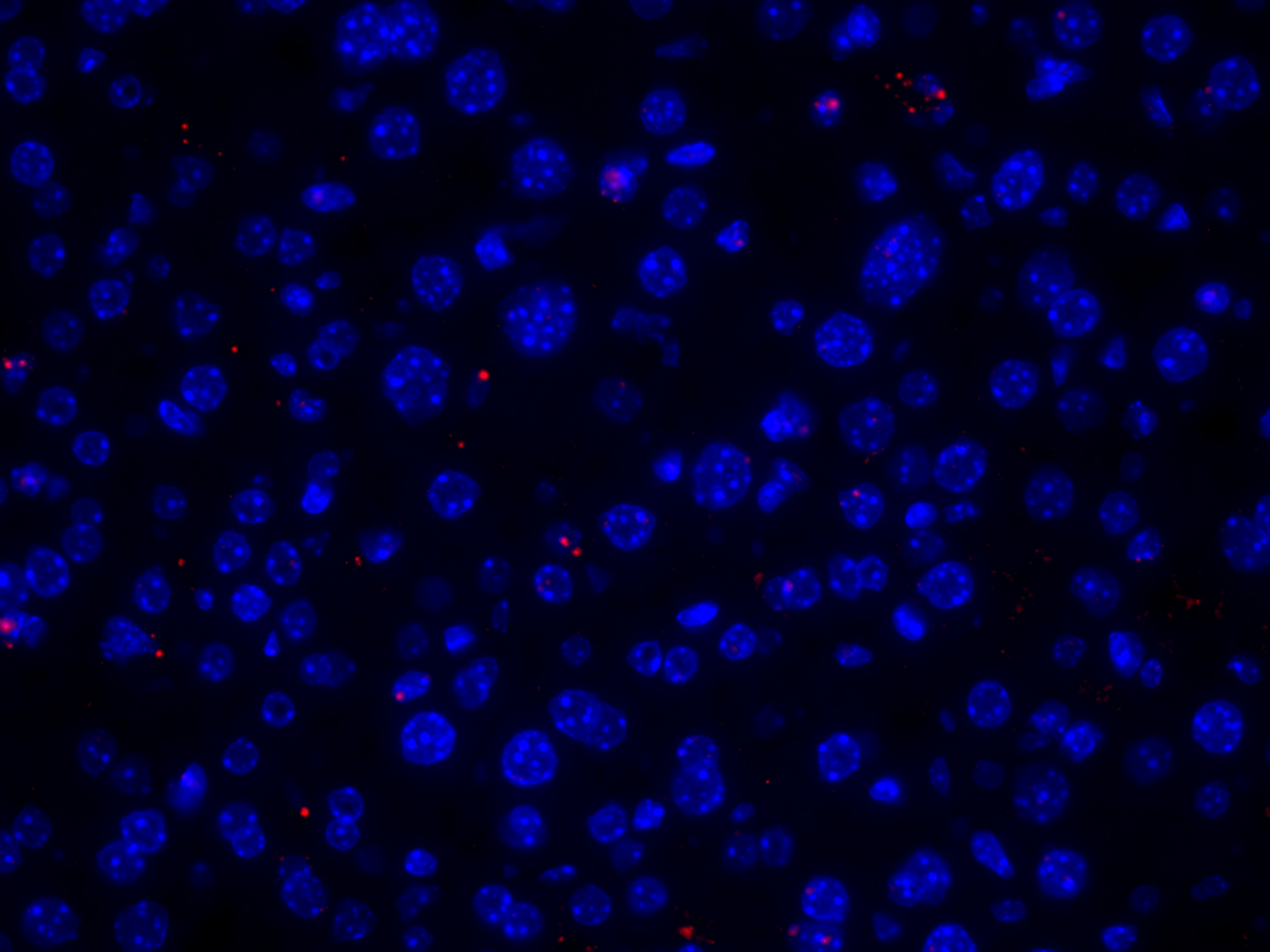

Supplement: Supplementary file 3 — Source data Fig. 2 [file 44321_2026_400_MOESM3_ESM.zip › Fig 2/2a/Liver_DJ900_tomatidine_p16_ select image 3.lif - Image 2.png]

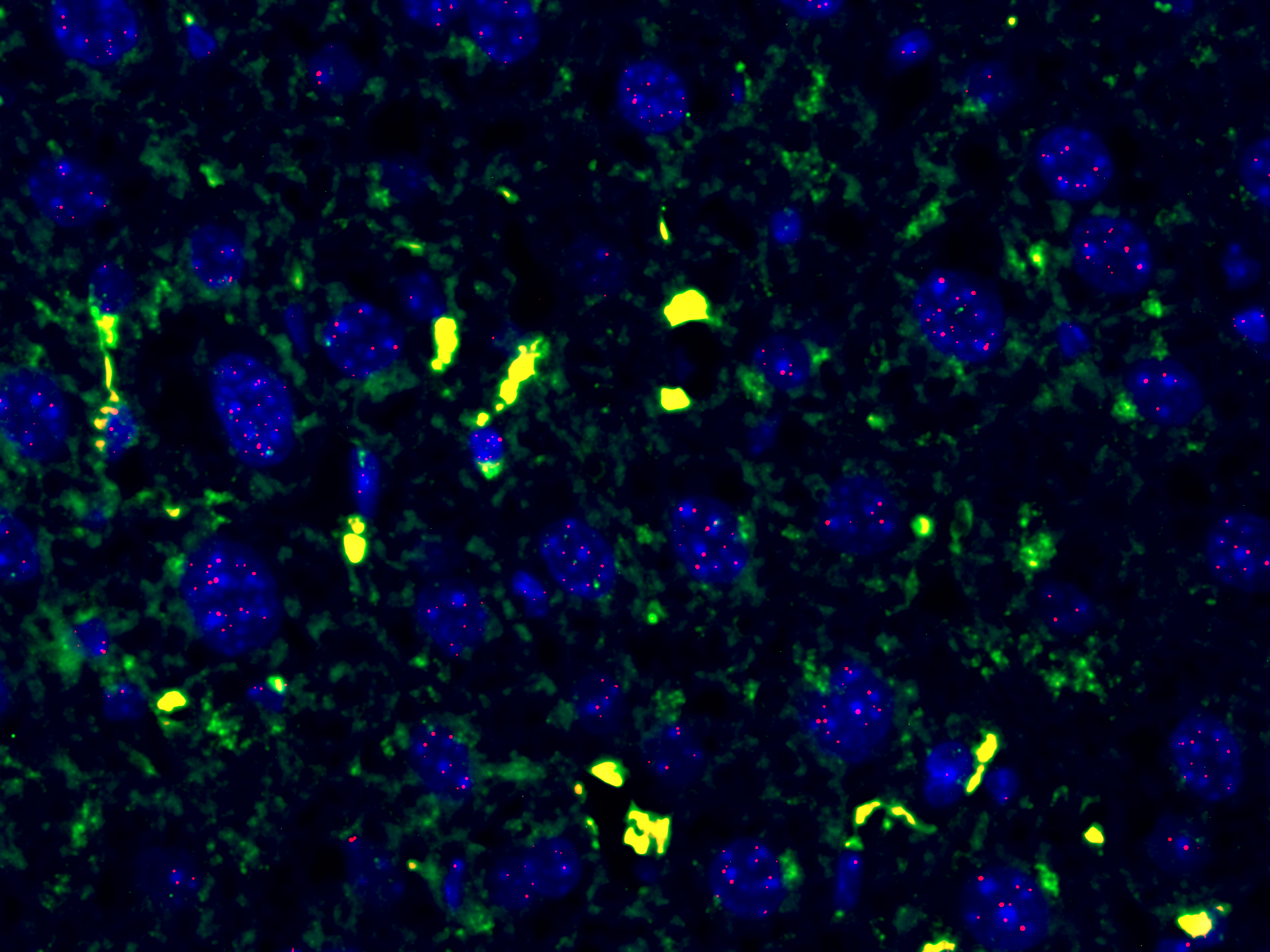

Supplement: Supplementary file 3 — Source data Fig. 2 [file 44321_2026_400_MOESM3_ESM.zip › Fig 2/2d/tomatidine SUM_TAF_DJ873_Liver_DGC_3.26.25.lif - Image 4 stack 9-37.png]

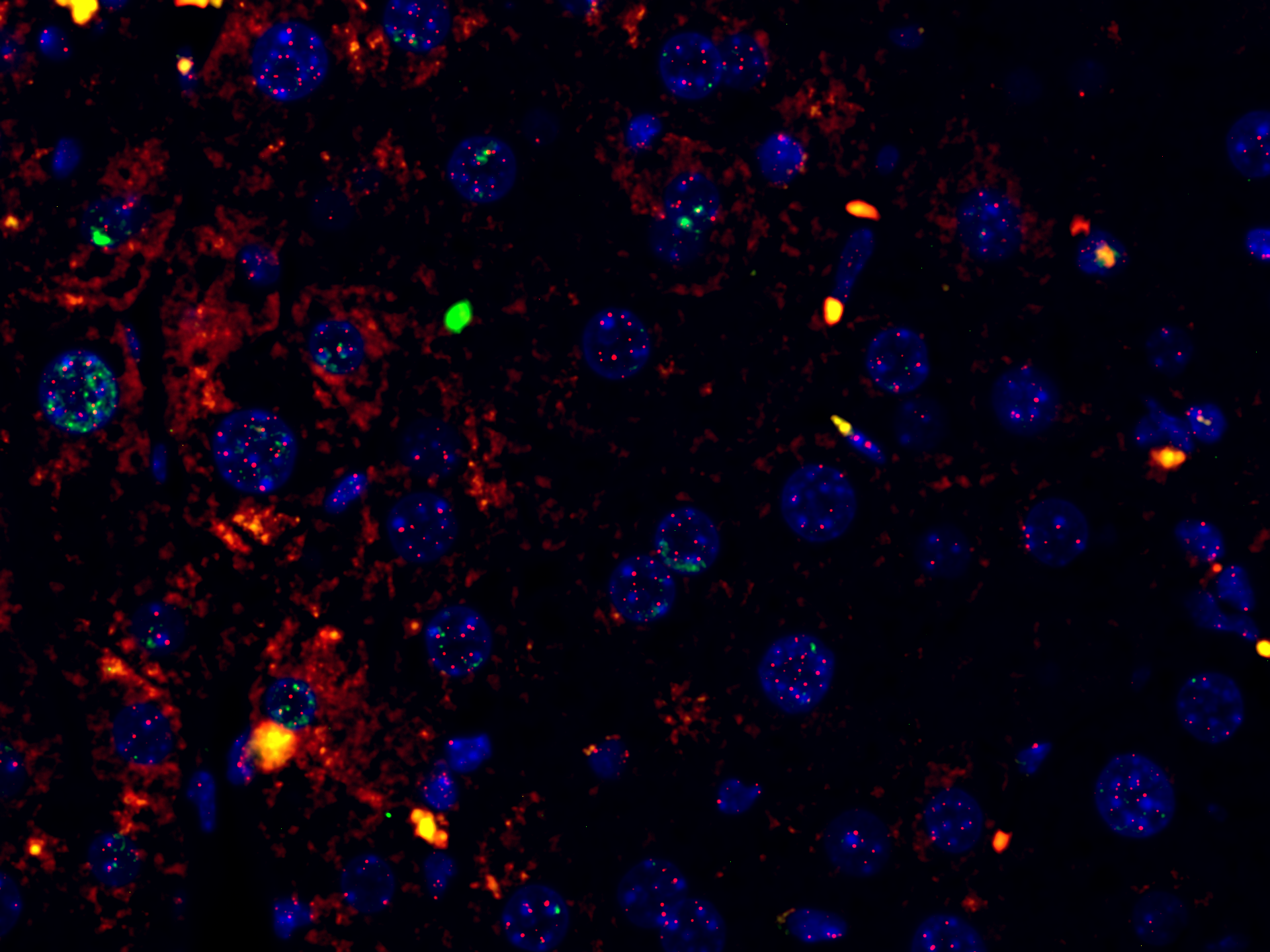

Supplement: Supplementary file 3 — Source data Fig. 2 [file 44321_2026_400_MOESM3_ESM.zip › Fig 2/2d/vehicle SUM_TAF_DJ942_Liver_DGC_3.26.25.lif - Image 1 stack 14-36.png]

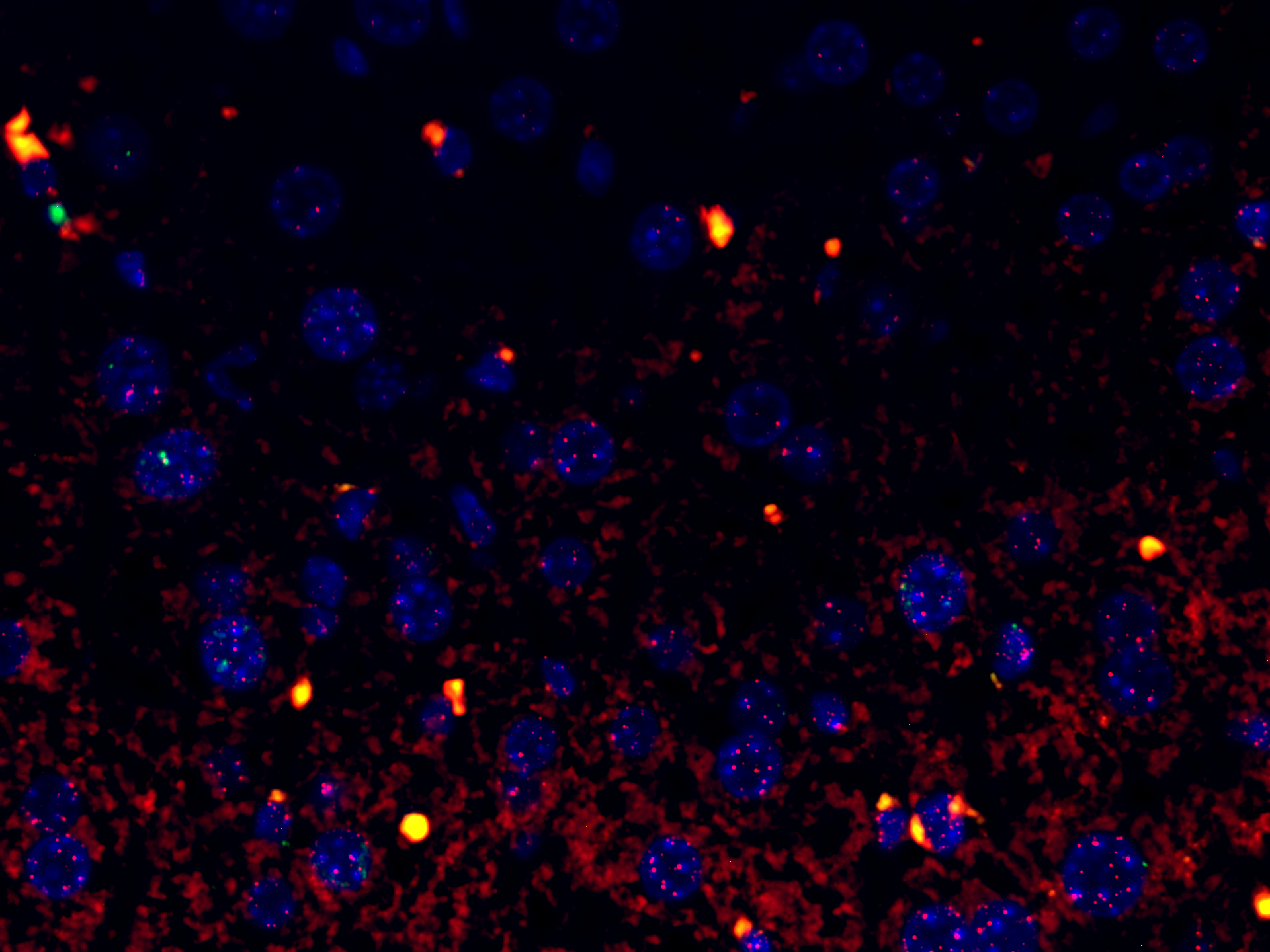

Supplement: Supplementary file 3 — Source data Fig. 2 [file 44321_2026_400_MOESM3_ESM.zip › Fig 2/2d/vehicle SUM_TAF_DJ942_Liver_DGC_3.26.25.lif - Image 3 stack 12-35.png]

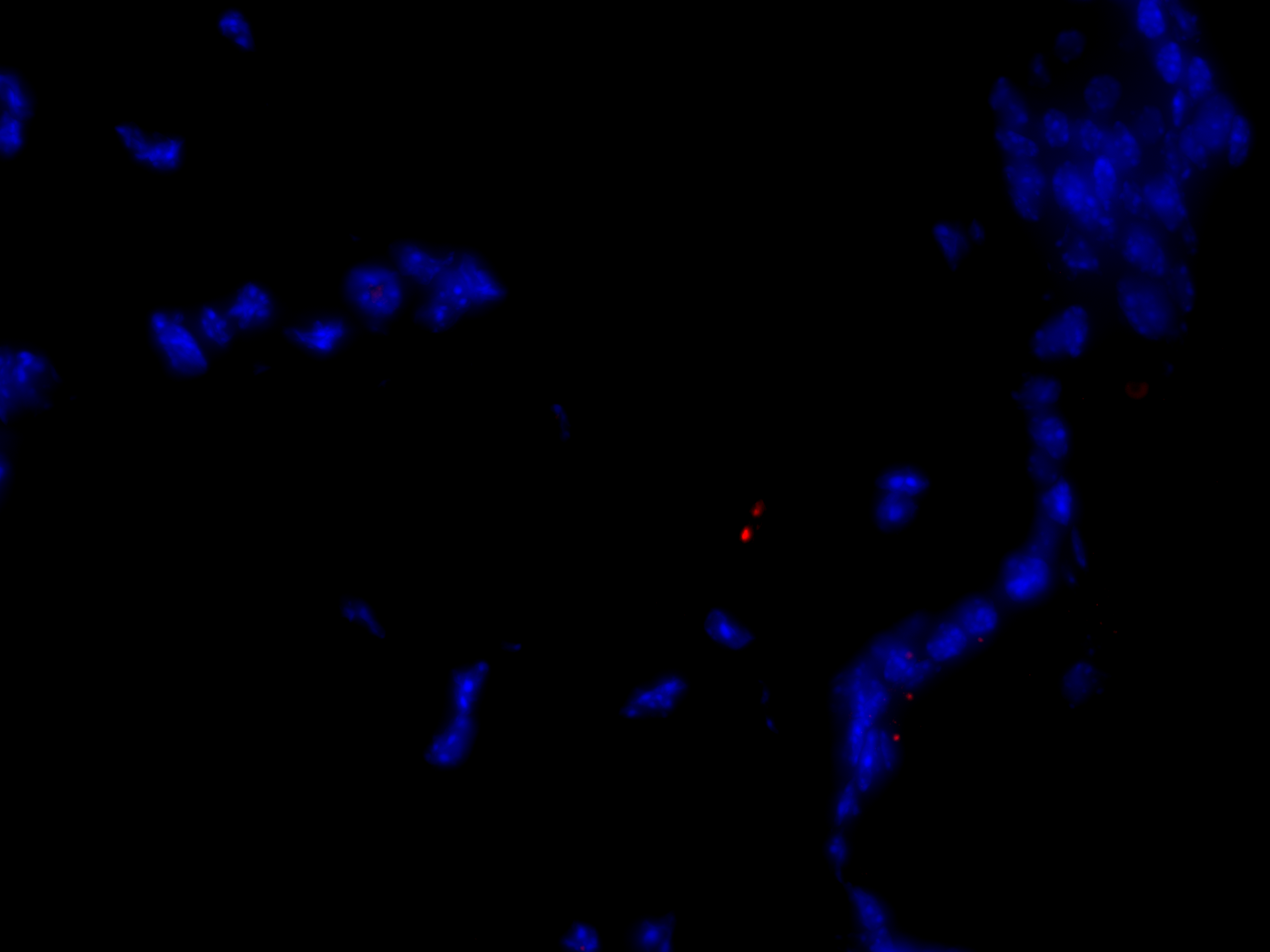

Supplement: Supplementary file 3 — Source data Fig. 2 [file 44321_2026_400_MOESM3_ESM.zip › Fig 2/2f/p16 RNAis DJ 896 tomatidine.png]

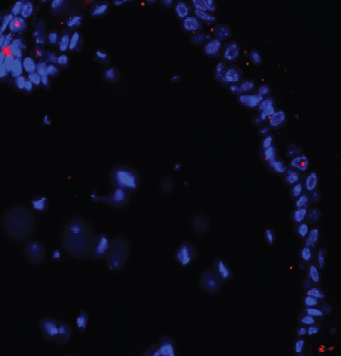

Supplement: Supplementary file 3 — Source data Fig. 2 [file 44321_2026_400_MOESM3_ESM.zip › Fig 2/2f/Vector Smart Object-1.png]

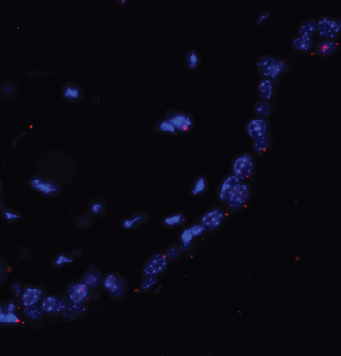

Supplement: Supplementary file 3 — Source data Fig. 2 [file 44321_2026_400_MOESM3_ESM.zip › Fig 2/2f/Vector Smart Object-2.png]

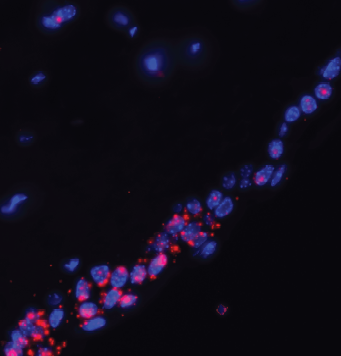

Supplement: Supplementary file 3 — Source data Fig. 2 [file 44321_2026_400_MOESM3_ESM.zip › Fig 2/2f/Vector Smart Object-3.png]

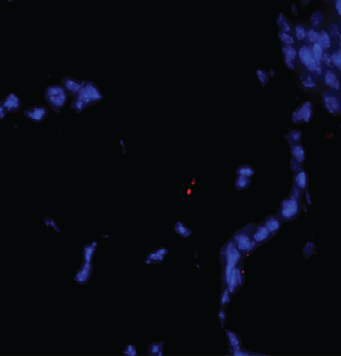

Supplement: Supplementary file 3 — Source data Fig. 2 [file 44321_2026_400_MOESM3_ESM.zip › Fig 2/2f/Vector Smart Object.png]

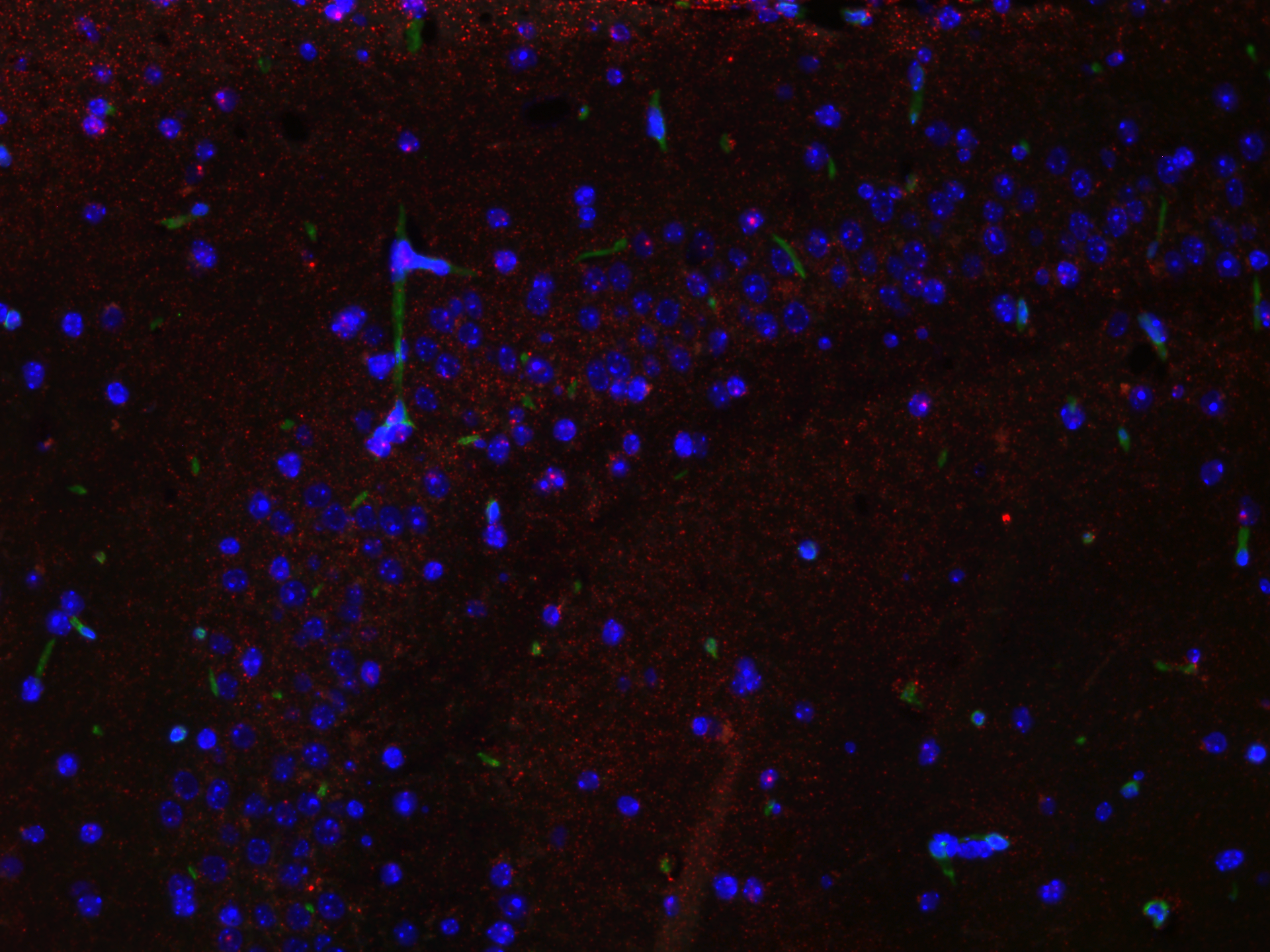

Supplement: Supplementary file 4 — Source data Fig. 3 [file 44321_2026_400_MOESM4_ESM.zip › Fig 3/Fig 3a/Hippocampus_DJ 875_Tomatidine_p21+Glut1_DGC.lif - Image 2.png]

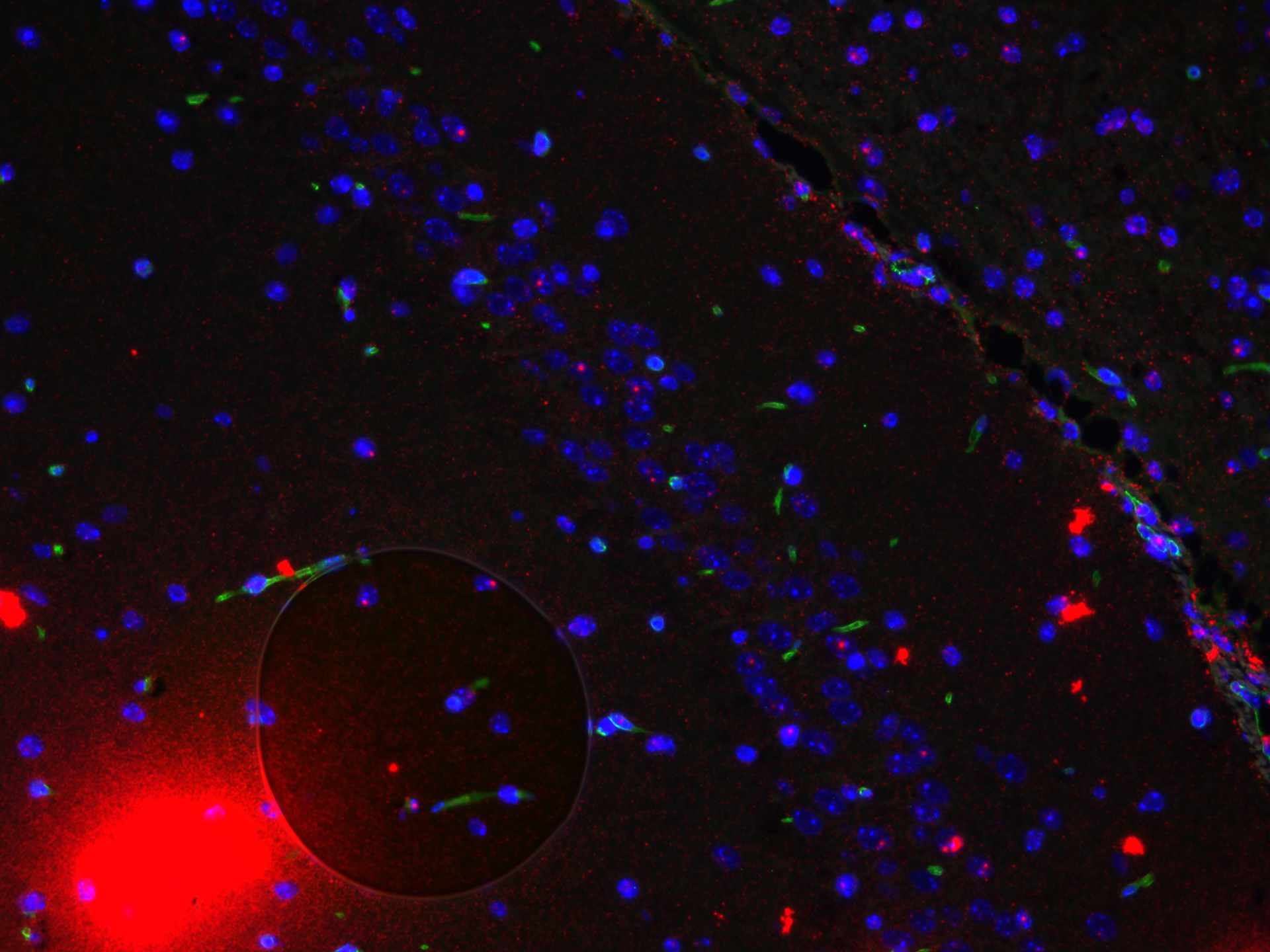

Supplement: Supplementary file 4 — Source data Fig. 3 [file 44321_2026_400_MOESM4_ESM.zip › Fig 3/Fig 3a/Hippocampus_DJ 942_vehicle_p21+Glut1_DGC.lif - Image 2.png]

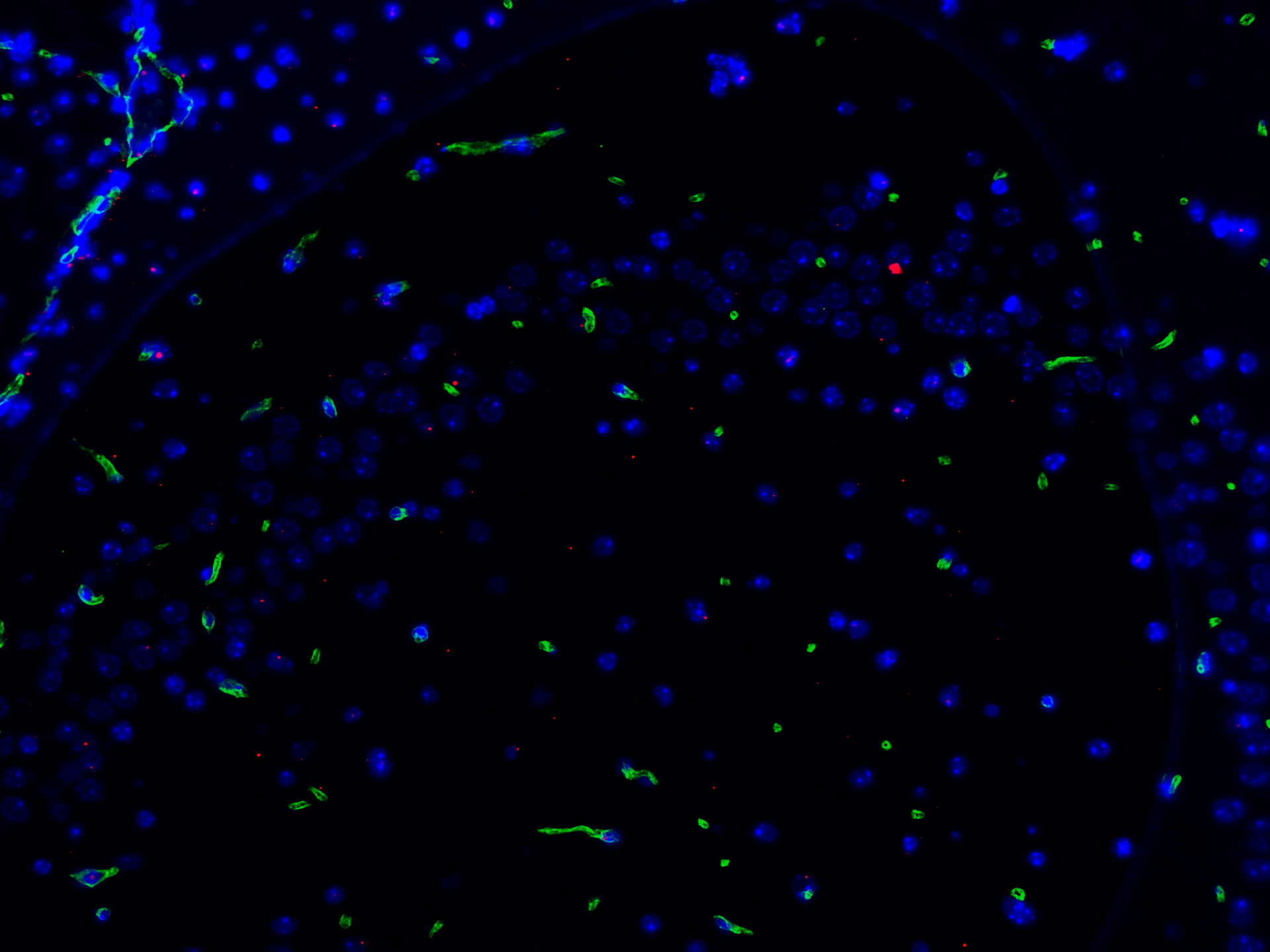

Supplement: Supplementary file 4 — Source data Fig. 3 [file 44321_2026_400_MOESM4_ESM.zip › Fig 3/Fig 3b/Hippocampus_DJ 879_Tomatidine_p16+Glut1_DGC.lif - Image 2.png]

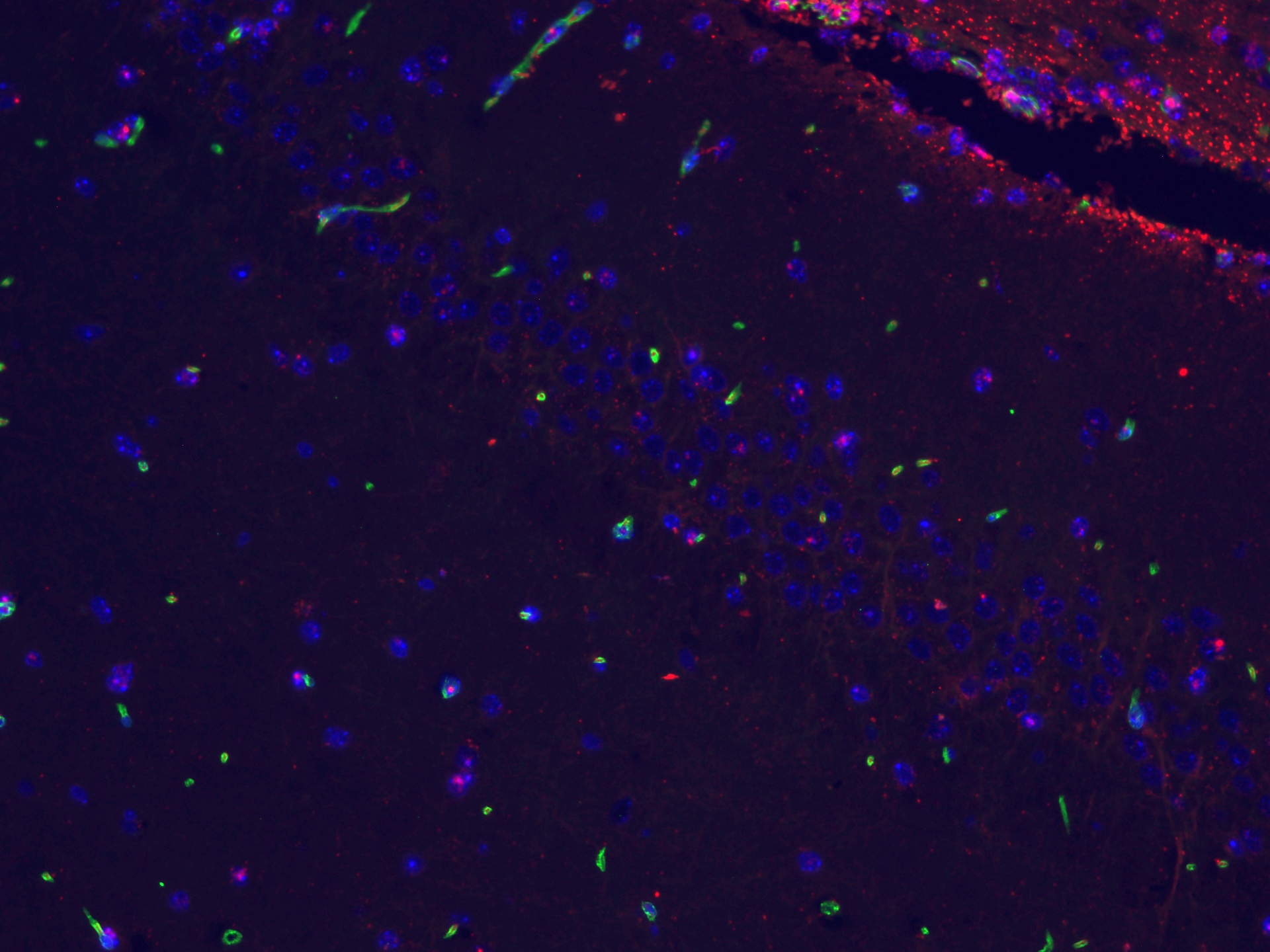

Supplement: Supplementary file 4 — Source data Fig. 3 [file 44321_2026_400_MOESM4_ESM.zip › Fig 3/Fig 3b/Hippocampus_DJ 941_vehicle_p16+Glut1_DGC.lif - Image 2.png]

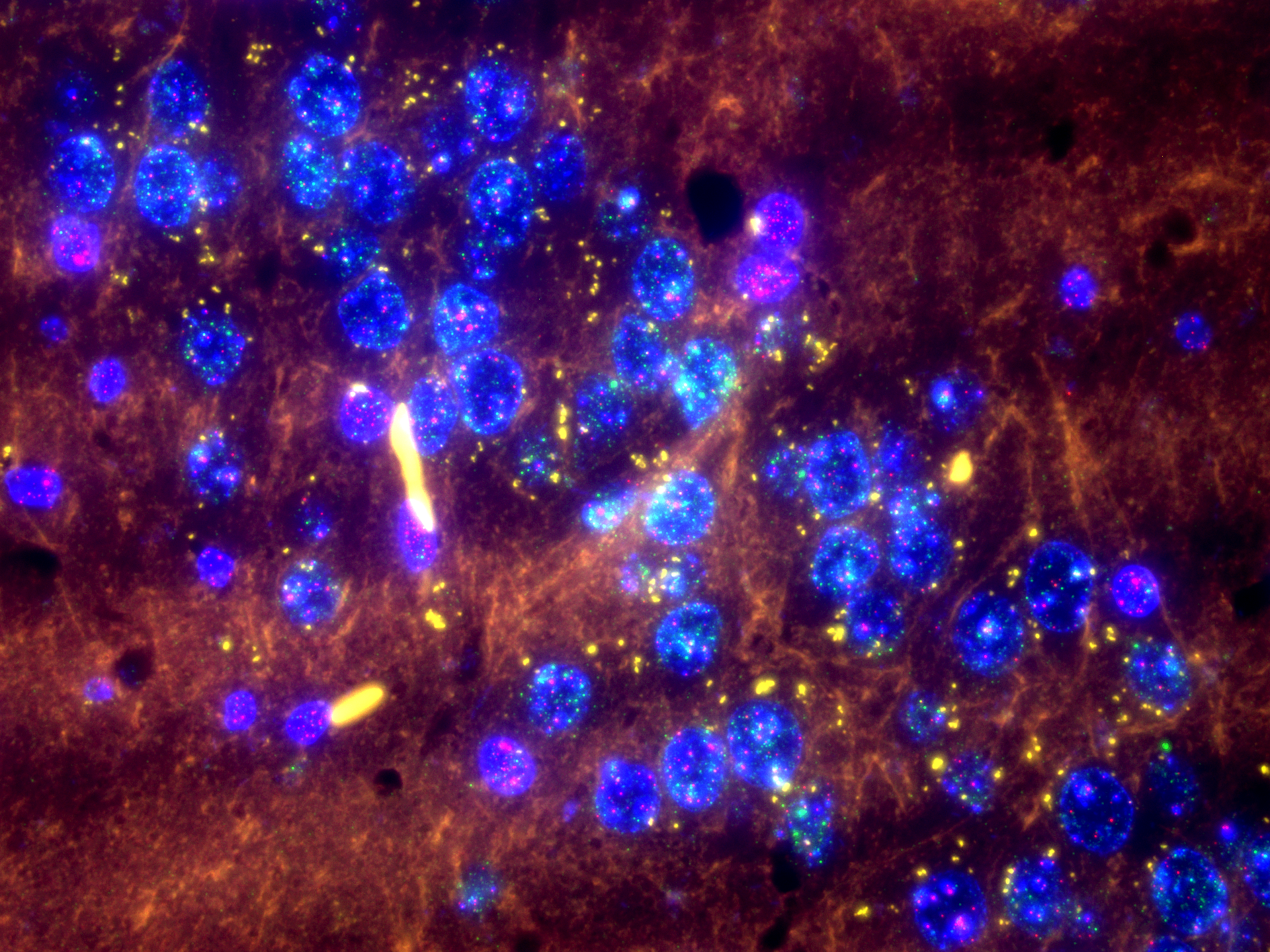

Supplement: Supplementary file 4 — Source data Fig. 3 [file 44321_2026_400_MOESM4_ESM.zip › Fig 3/Fig 3c/TAF brain vehicle 2.tif]

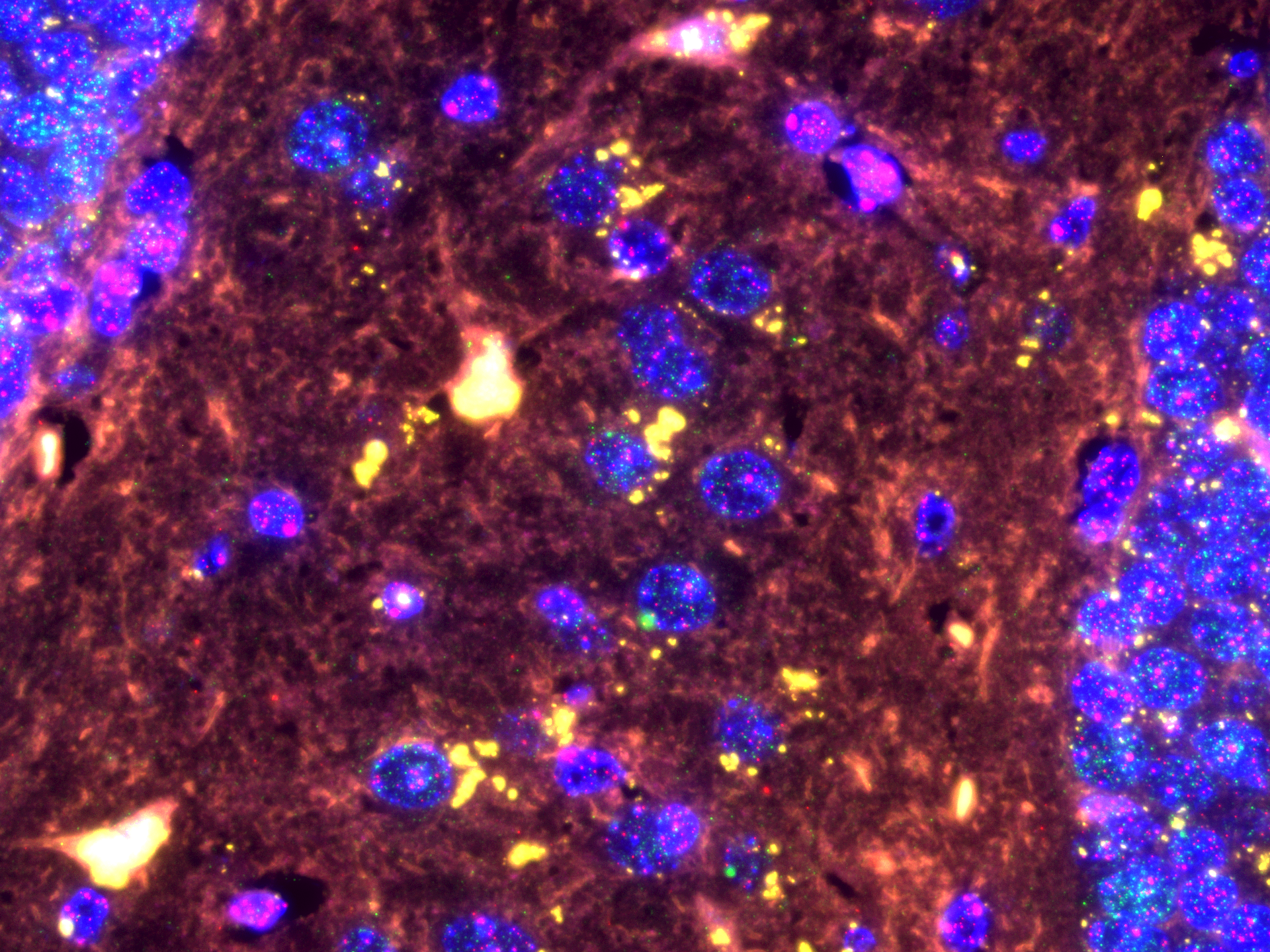

Supplement: Supplementary file 4 — Source data Fig. 3 [file 44321_2026_400_MOESM4_ESM.zip › Fig 3/Fig 3c/TAF_MAX_Hippo_Tomatidine_Animal 879_DGC.lif - Image 1 (RGB).tif]

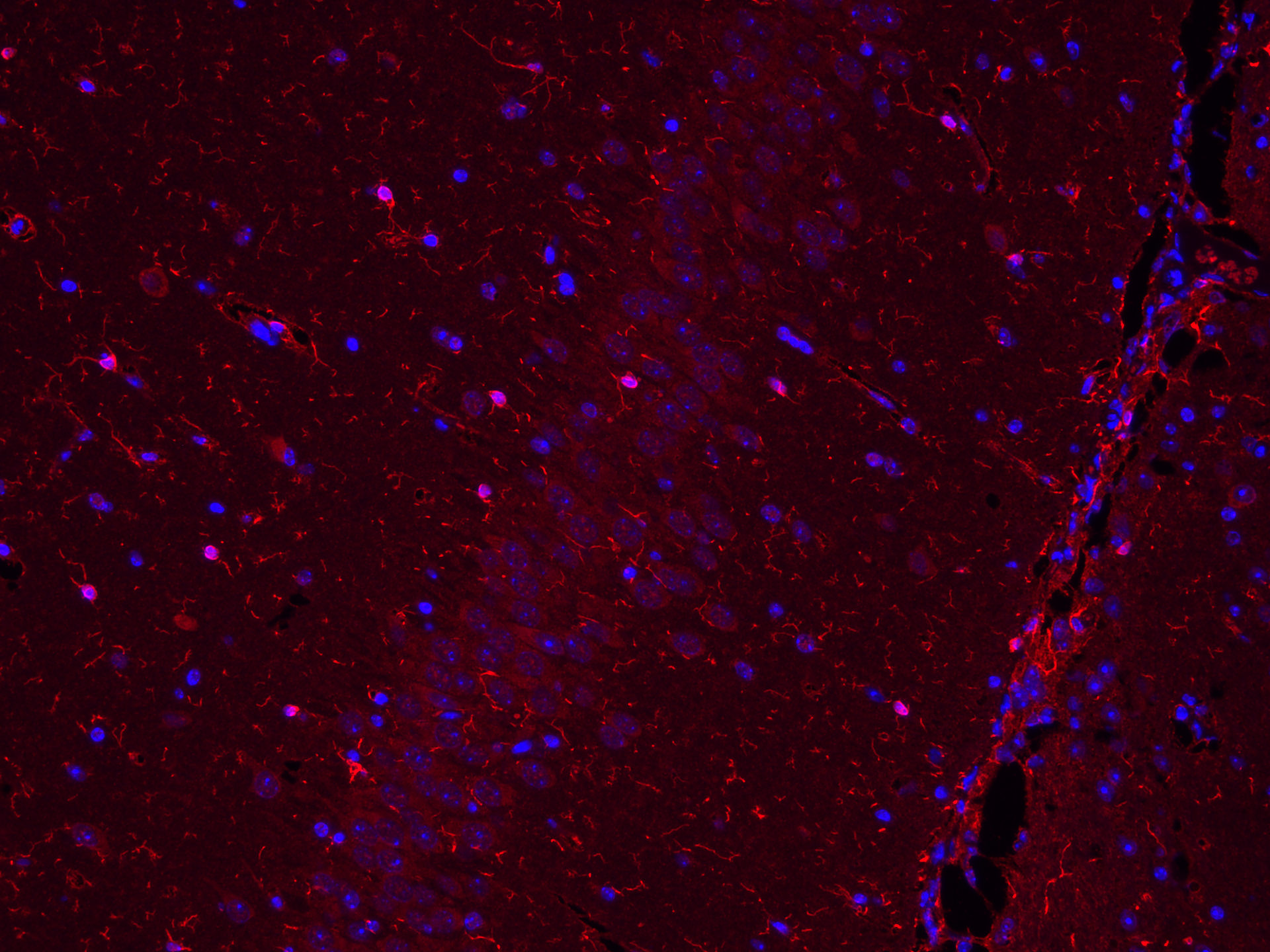

Supplement: Supplementary file 4 — Source data Fig. 3 [file 44321_2026_400_MOESM4_ESM.zip › Fig 3/Fig 3d/iBA1_DJ863_HIPPO_vehicle_DGC_4.6.25.lif - Image 3.png]

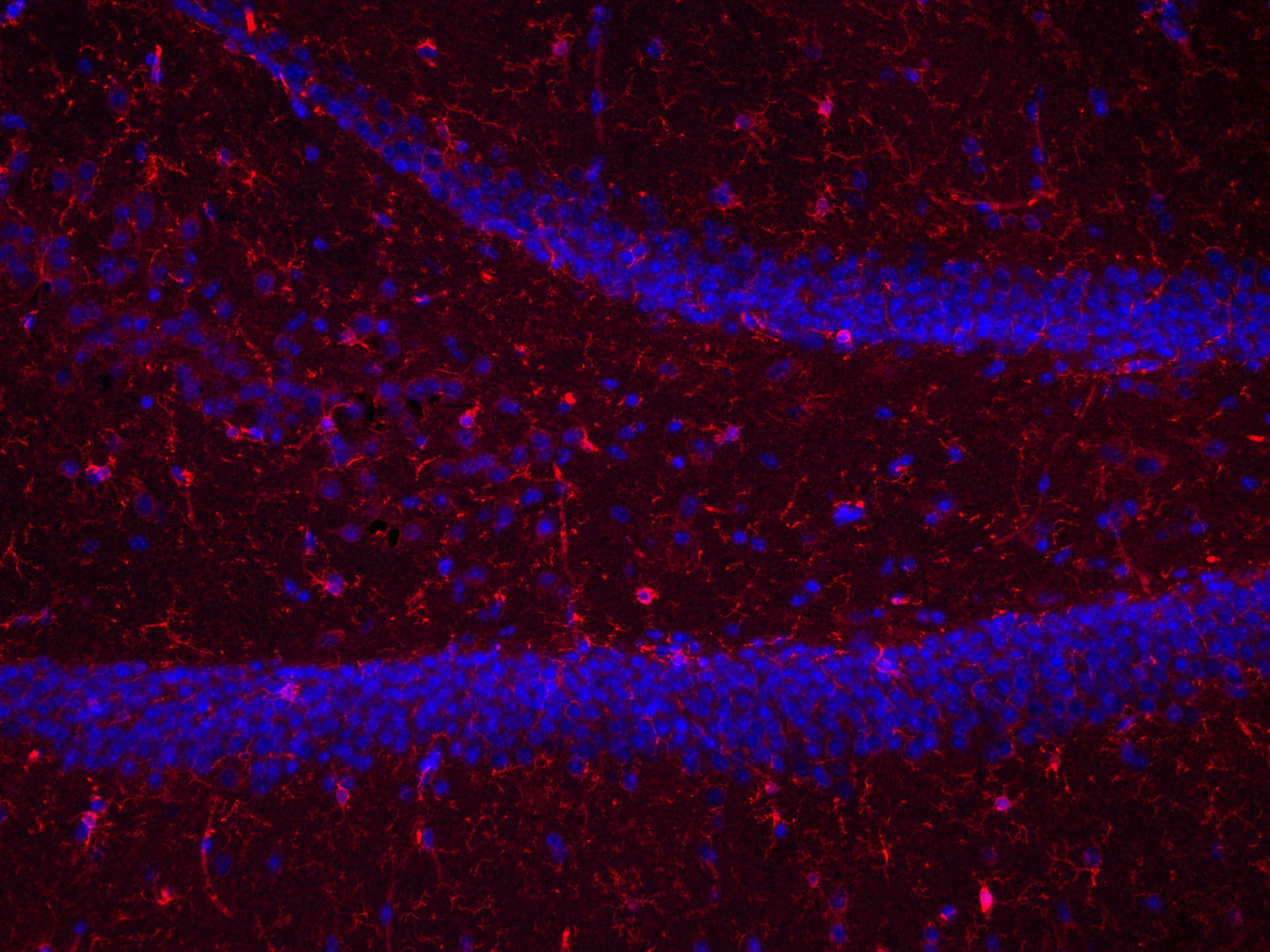

Supplement: Supplementary file 4 — Source data Fig. 3 [file 44321_2026_400_MOESM4_ESM.zip › Fig 3/Fig 3e/DJ862_IBA1.png]

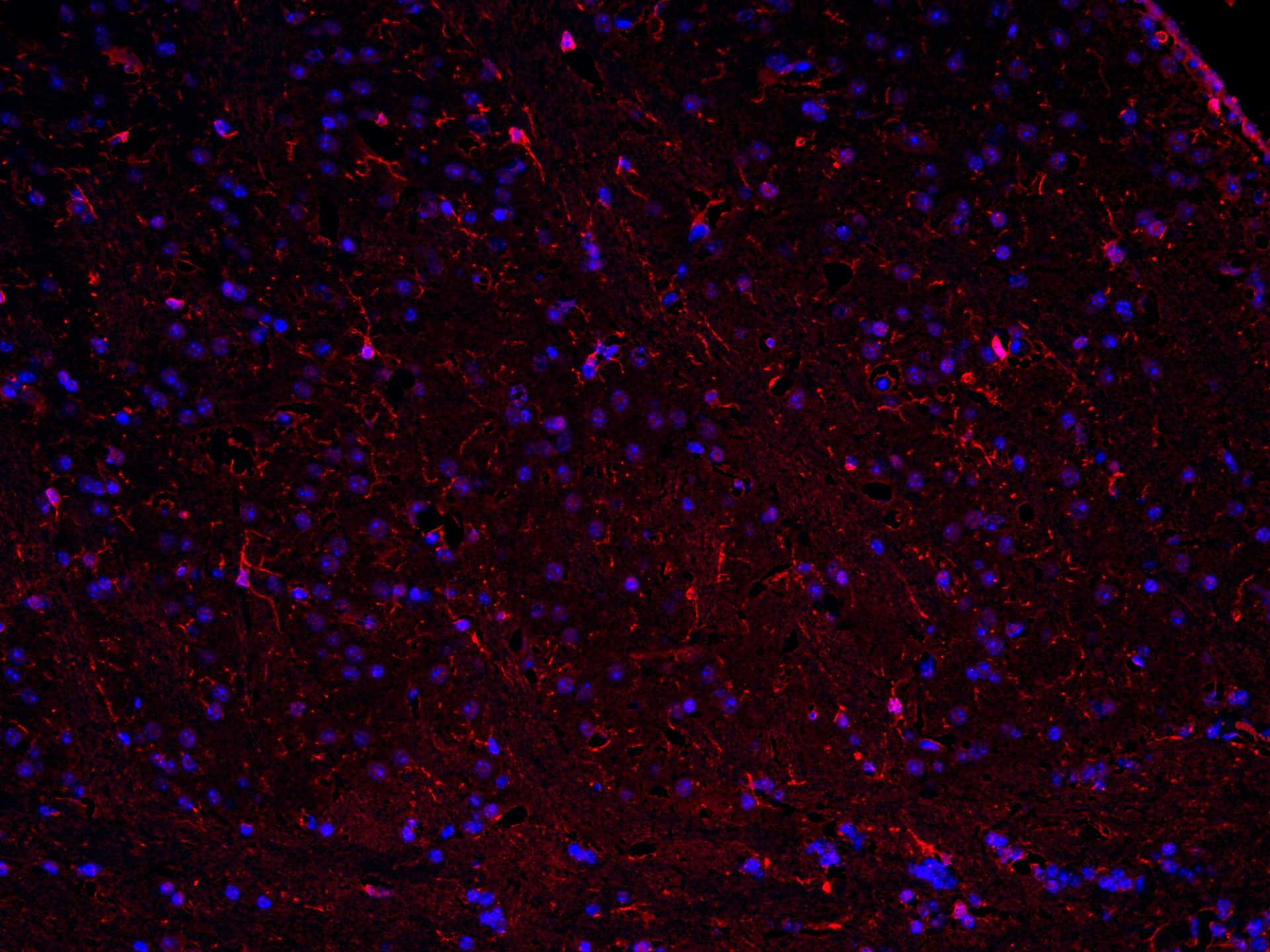

Supplement: Supplementary file 4 — Source data Fig. 3 [file 44321_2026_400_MOESM4_ESM.zip › Fig 3/Fig 3e/DJ866_IBA1_BBB_DGC_5 march 2025.lif - Image 1.png]

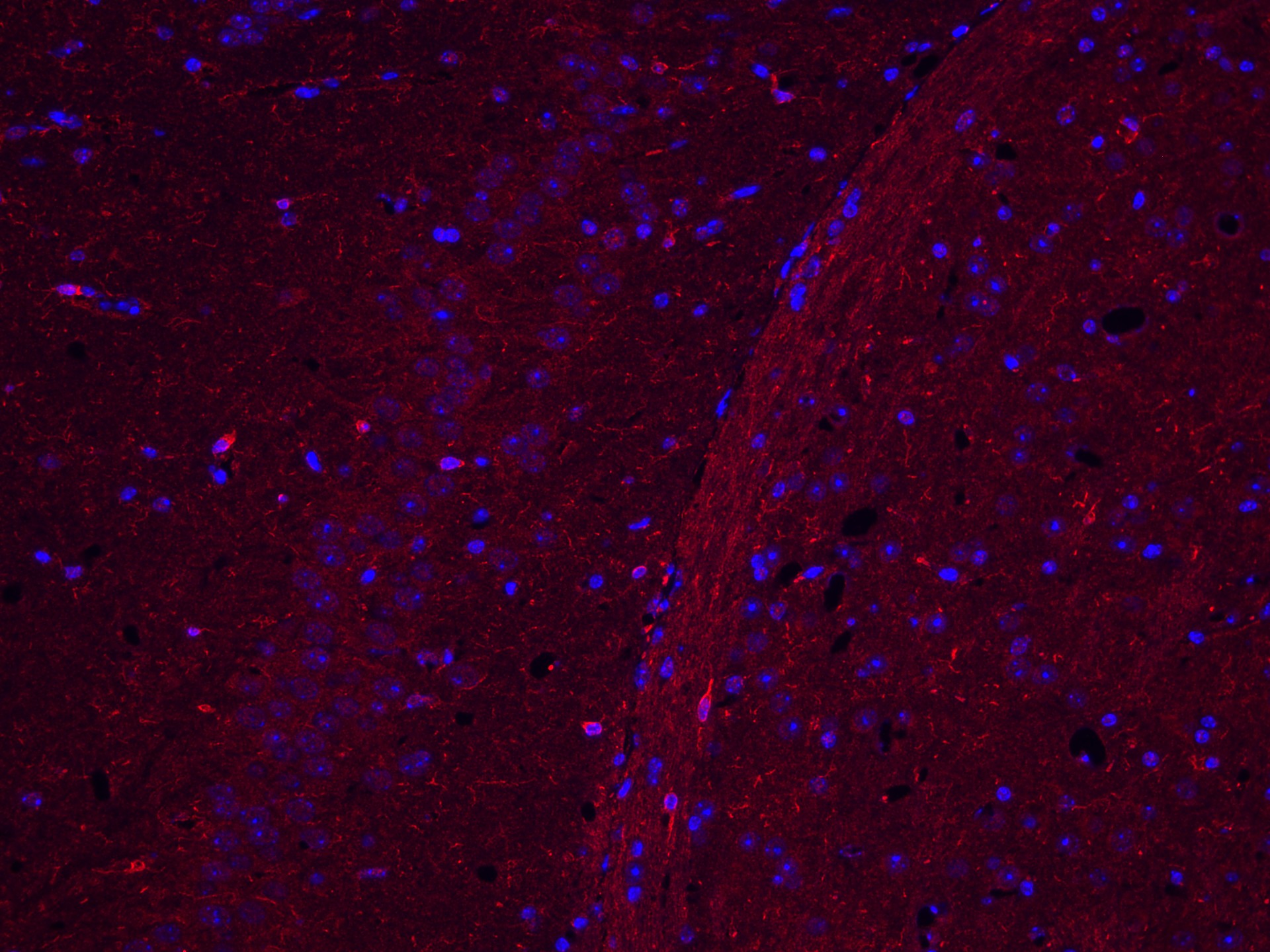

Supplement: Supplementary file 4 — Source data Fig. 3 [file 44321_2026_400_MOESM4_ESM.zip › Fig 3/Fig 3d/iBA1_DJ877_HIPPO_Tomatidine_DGC_4.6.25.lif - Image 2.jpg]

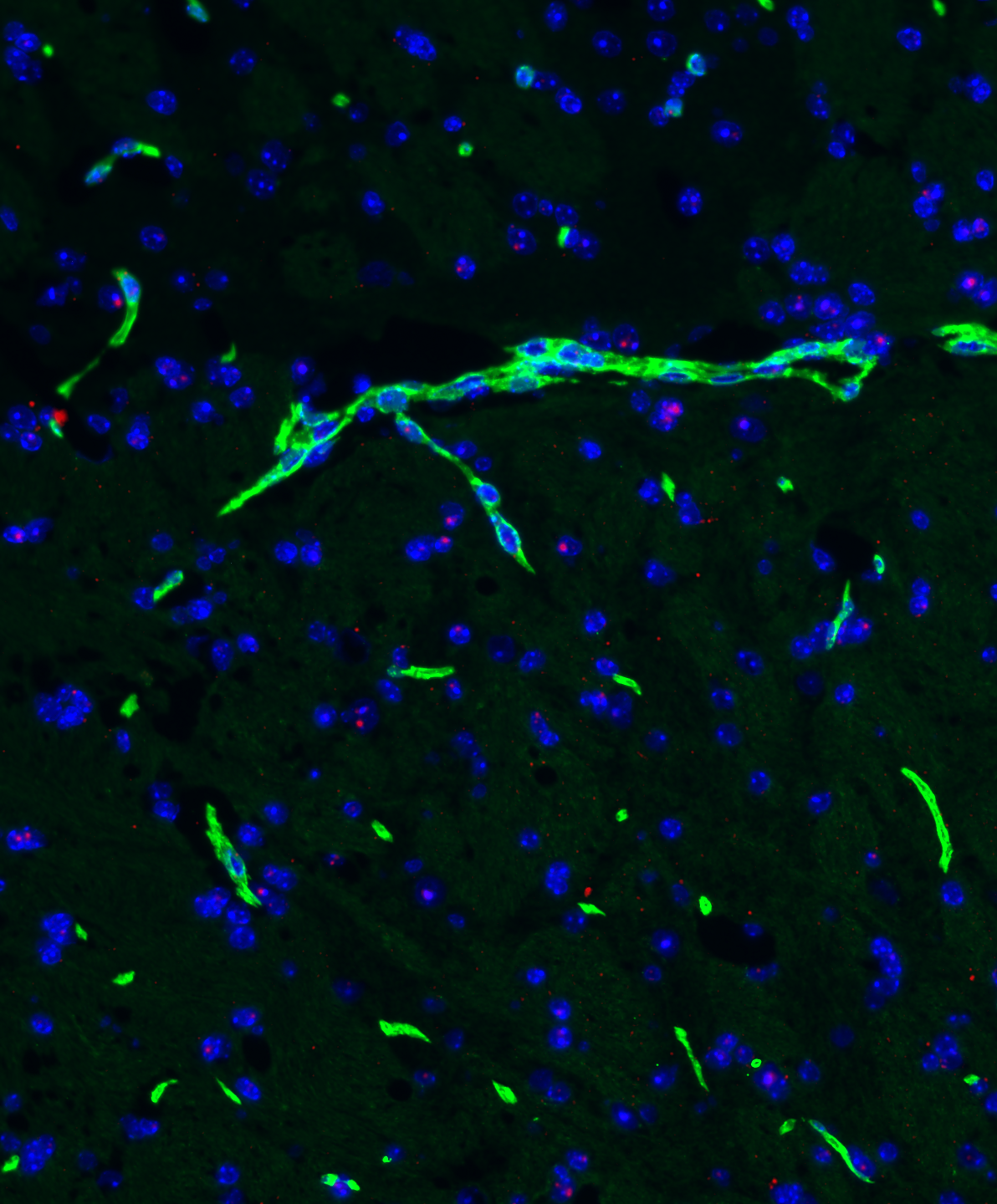

Supplement: Supplementary file 5 — Source data Fig. 4 [file 44321_2026_400_MOESM5_ESM.zip › Fig 4/Fig 4b/BBB_DJ 885_Tomatidine_p21+Glu1_DGC.lif - Image 2.png]

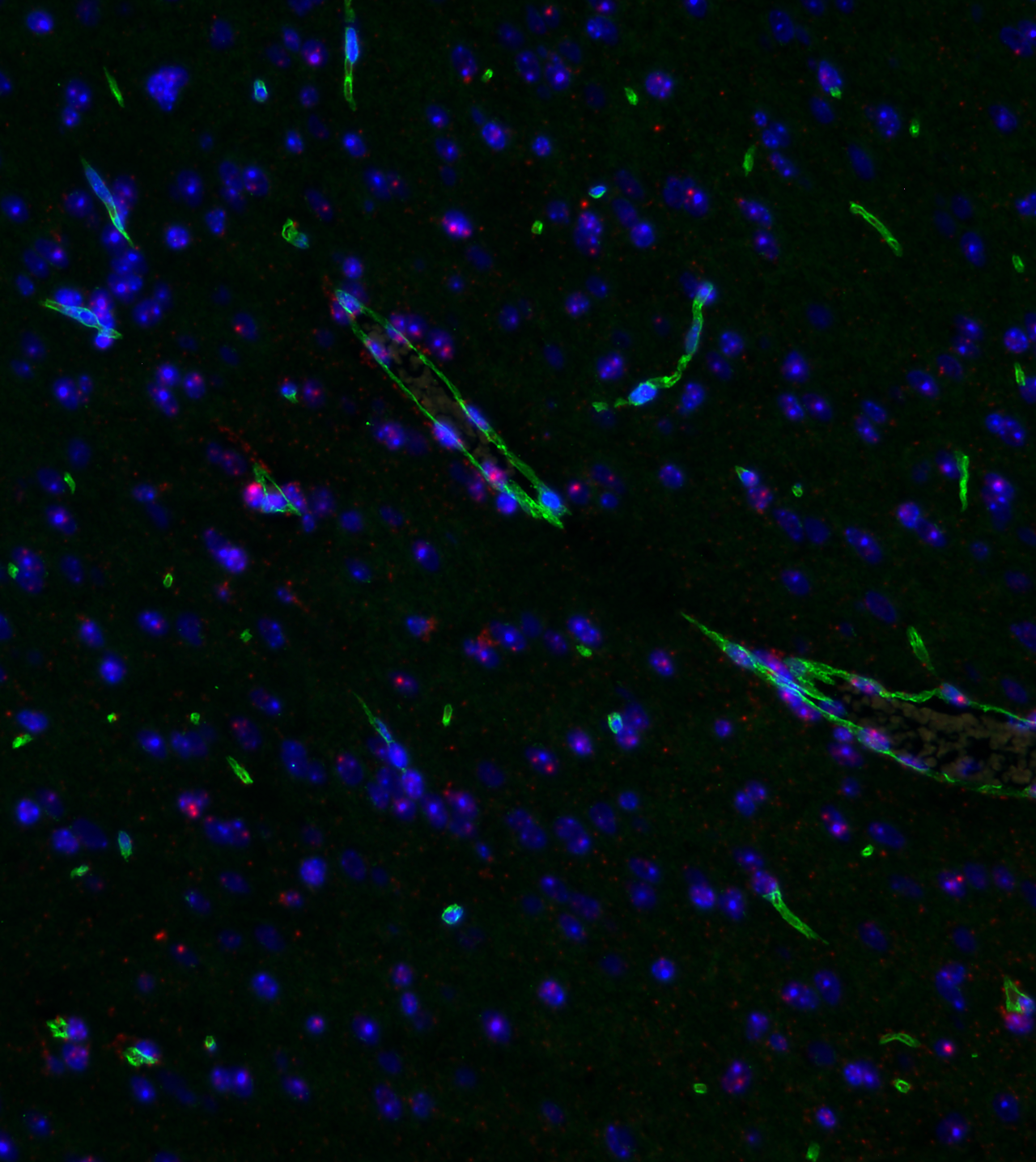

Supplement: Supplementary file 5 — Source data Fig. 4 [file 44321_2026_400_MOESM5_ESM.zip › Fig 4/Fig 4b/BBB_DJ 935_Tomatidine_p21+Glut1_DGC_vehicle treated.lif - Image 4.png]

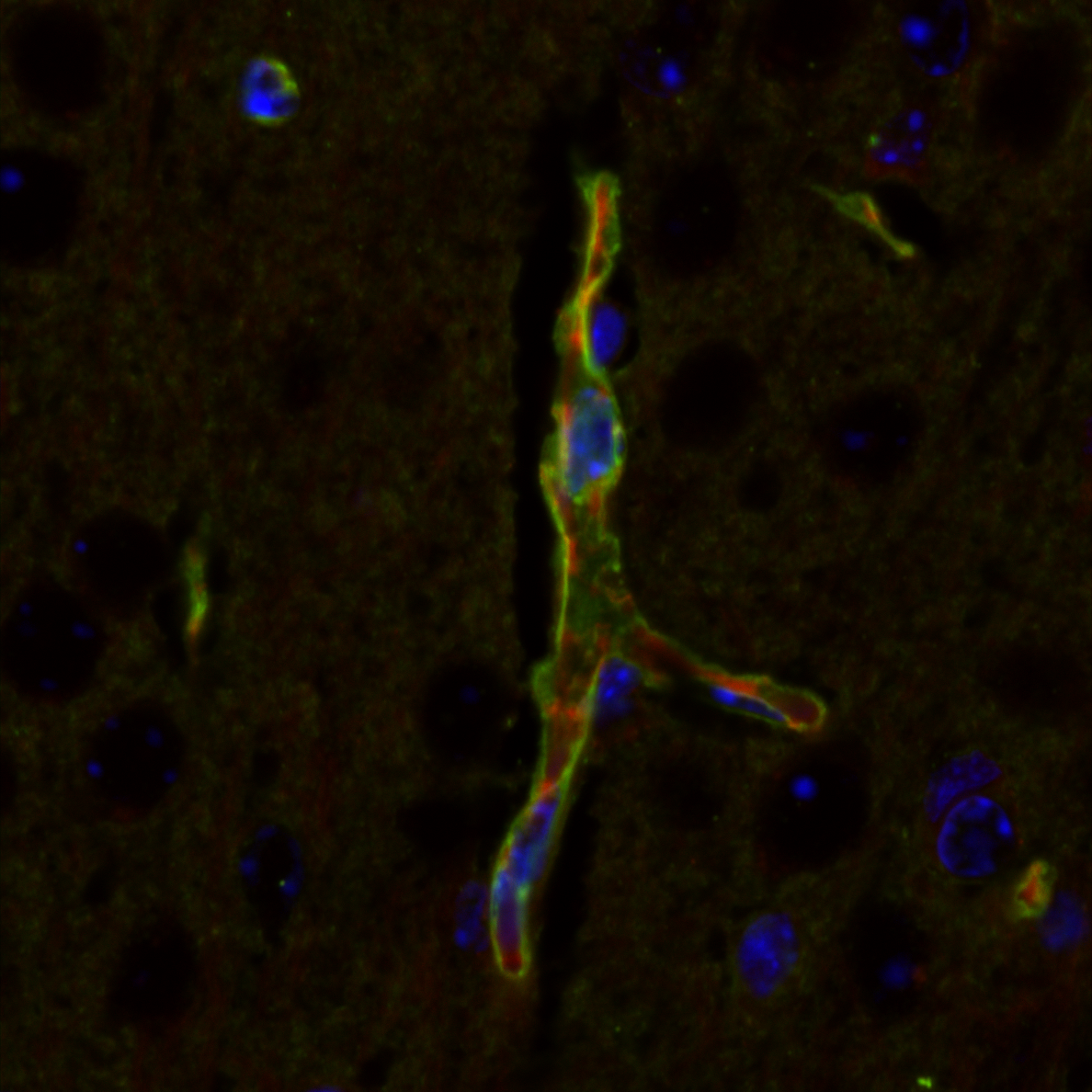

Supplement: Supplementary file 5 — Source data Fig. 4 [file 44321_2026_400_MOESM5_ESM.zip › Fig 4/Fig 4e/toma866-2 tomatidine ZO-1.png]

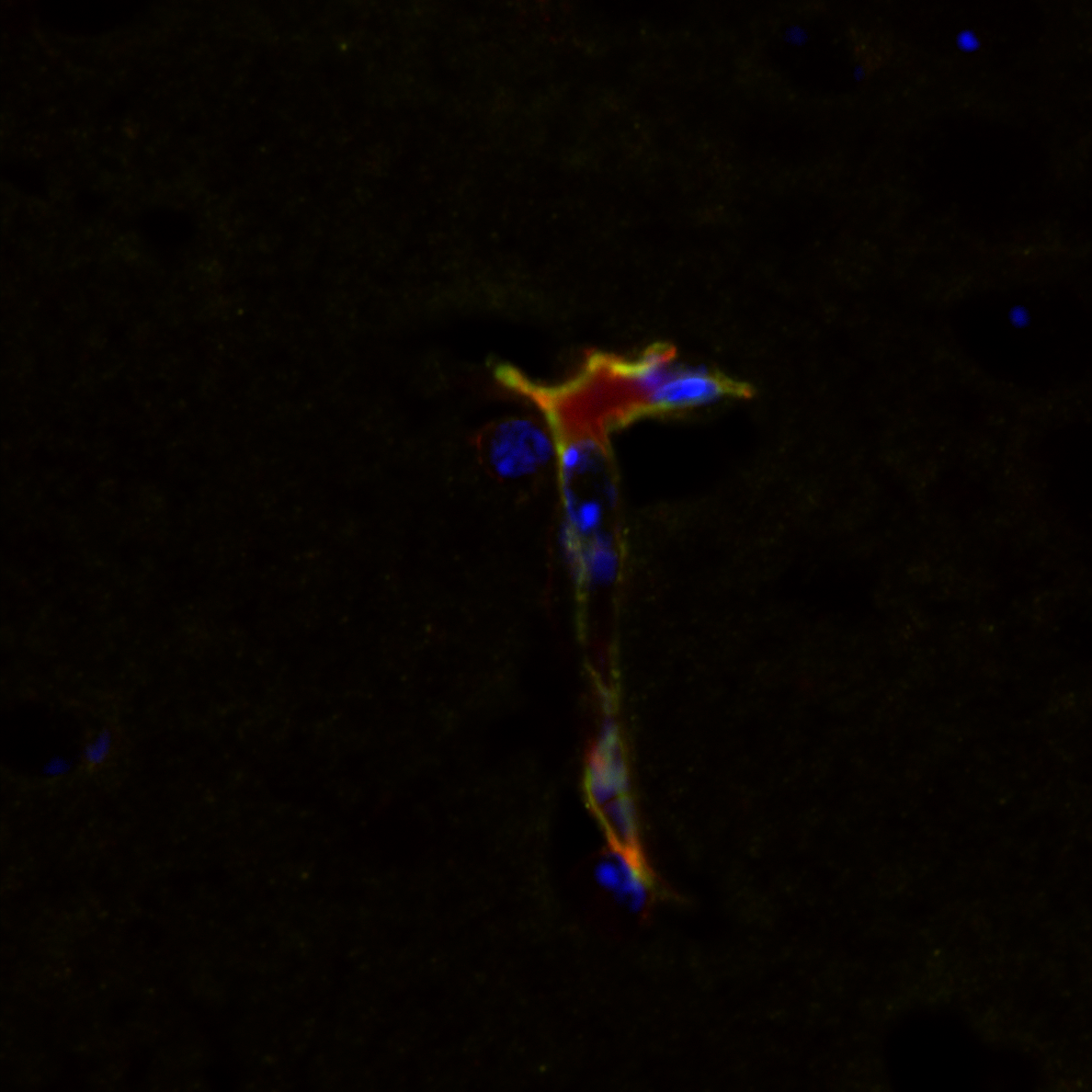

Supplement: Supplementary file 5 — Source data Fig. 4 [file 44321_2026_400_MOESM5_ESM.zip › Fig 4/Fig 4e/veh correct 865.png]

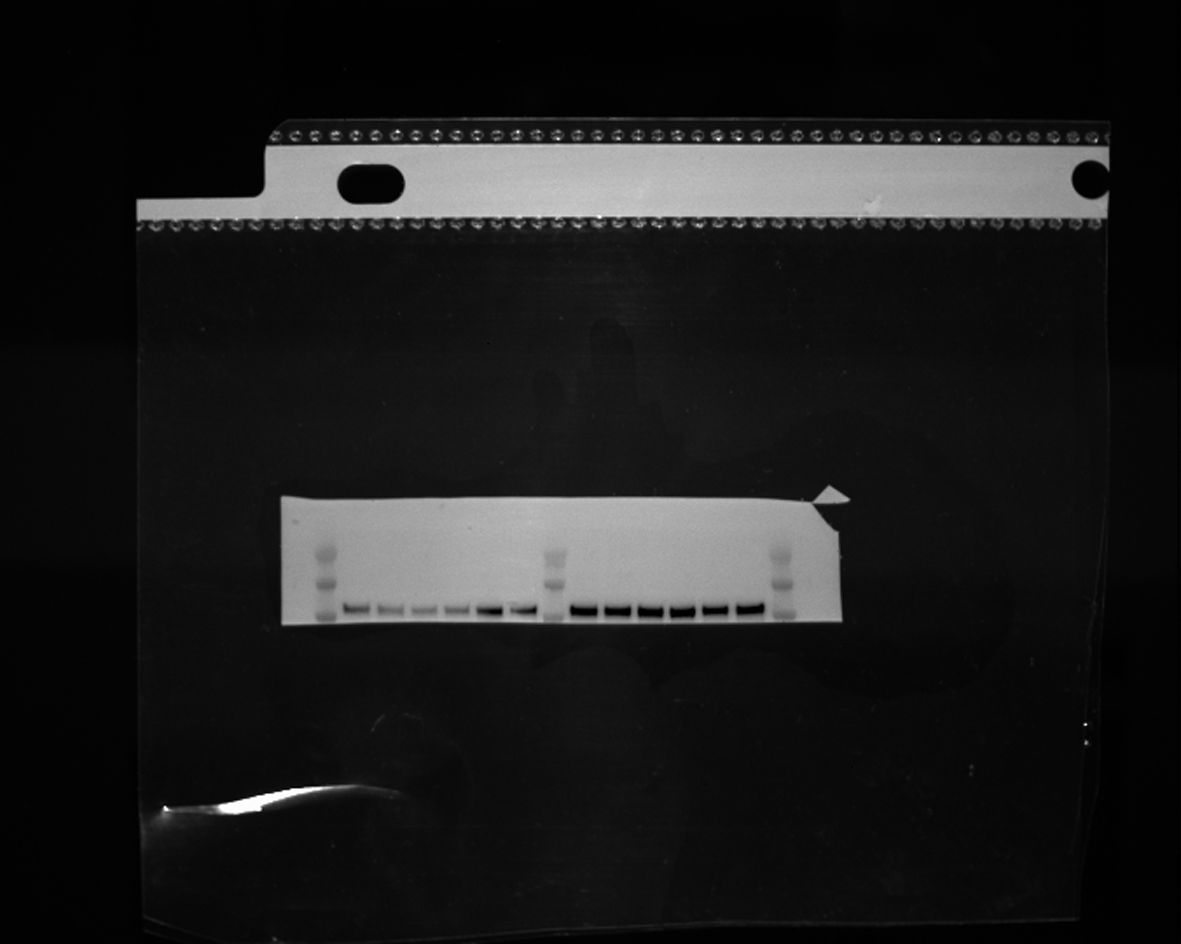

Supplement: Supplementary file 5 — Source data Fig. 4 [file 44321_2026_400_MOESM5_ESM.zip › Fig 4/Fig 4g/CHEMI_04302025_143720_(Chemi) ve-cadherin.tif]

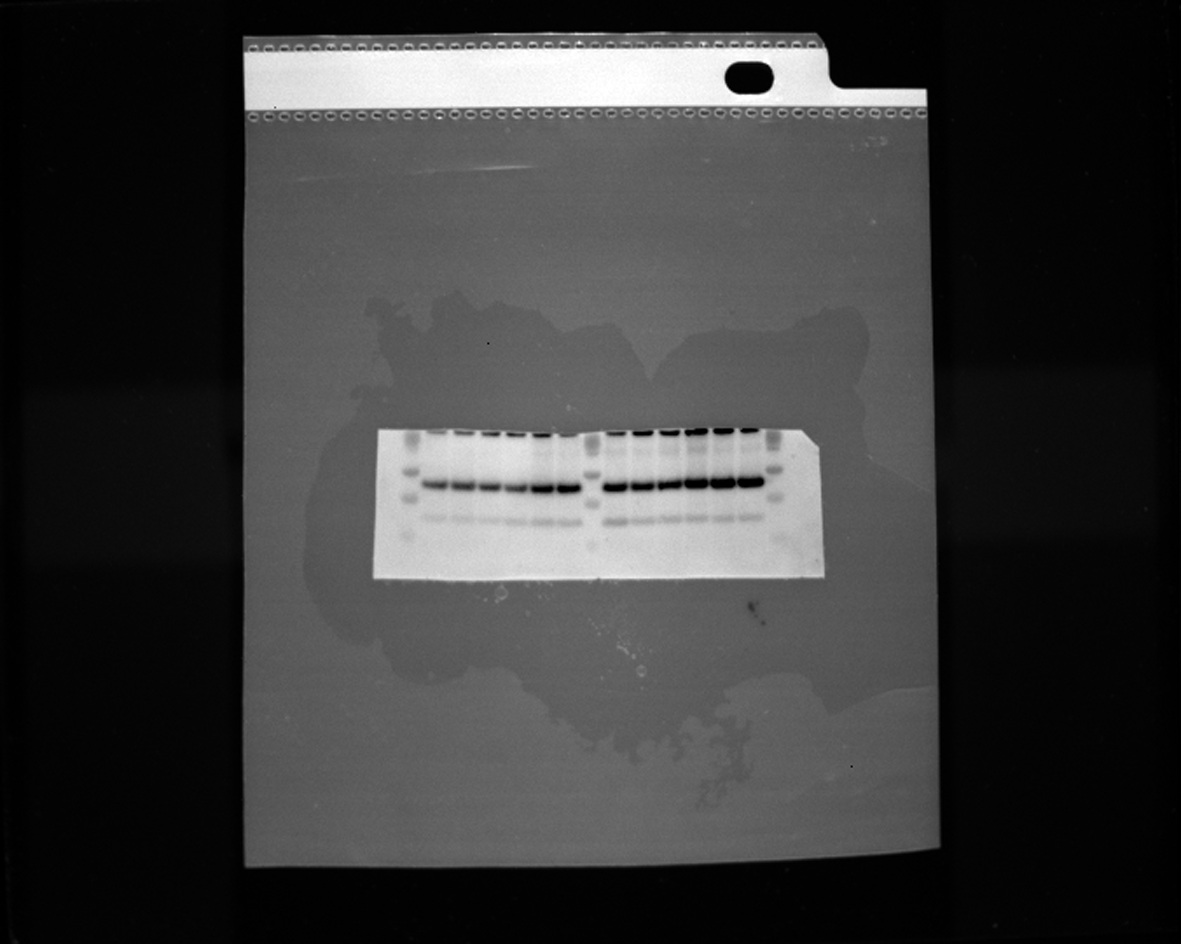

Supplement: Supplementary file 5 — Source data Fig. 4 [file 44321_2026_400_MOESM5_ESM.zip › Fig 4/Fig 4g/CHEMI_04302025_150105_(Chemi) Claudin 5.tif]

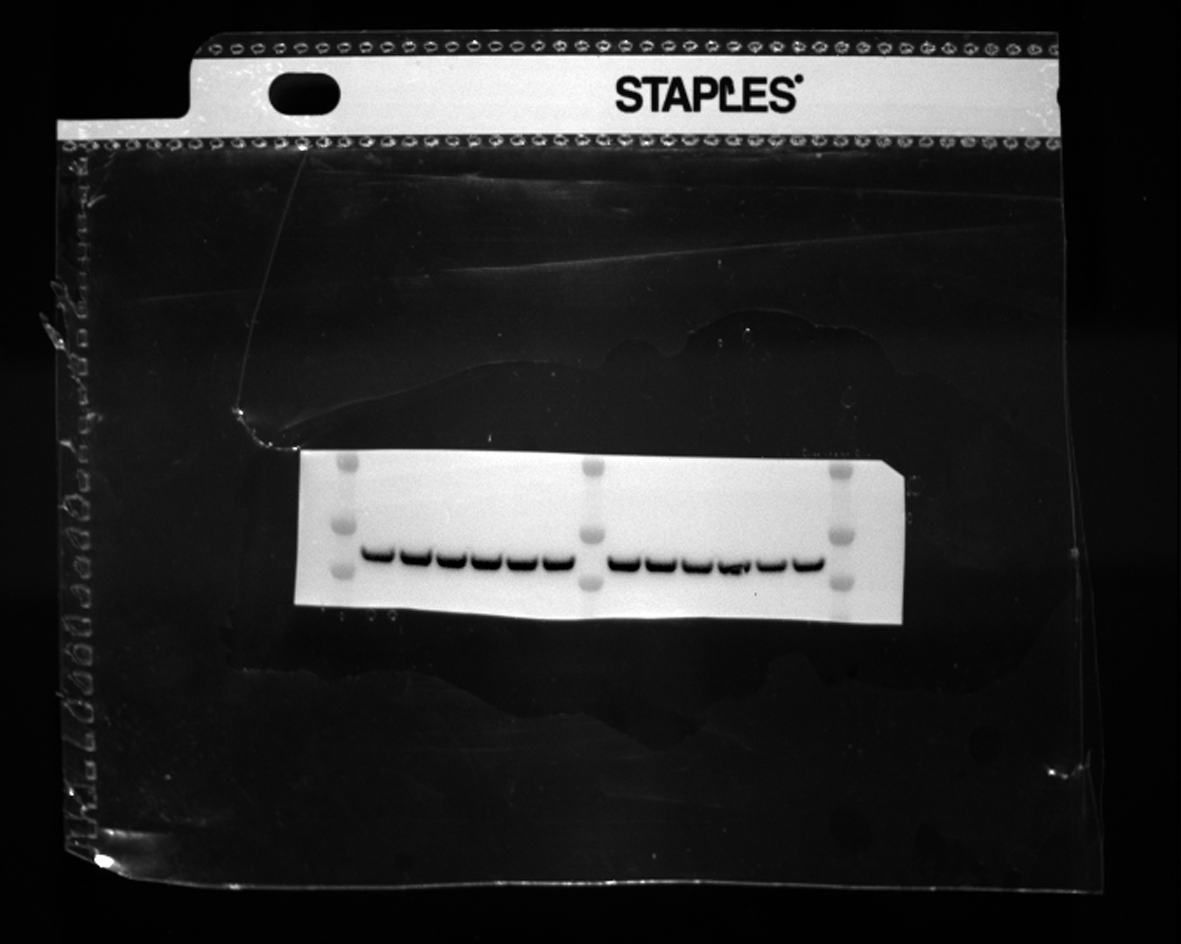

Supplement: Supplementary file 5 — Source data Fig. 4 [file 44321_2026_400_MOESM5_ESM.zip › Fig 4/Fig 4g/CHEMI_05012025_145829_(Chemi) ACTIN for claudin 5 and ve-cadherin.tif]

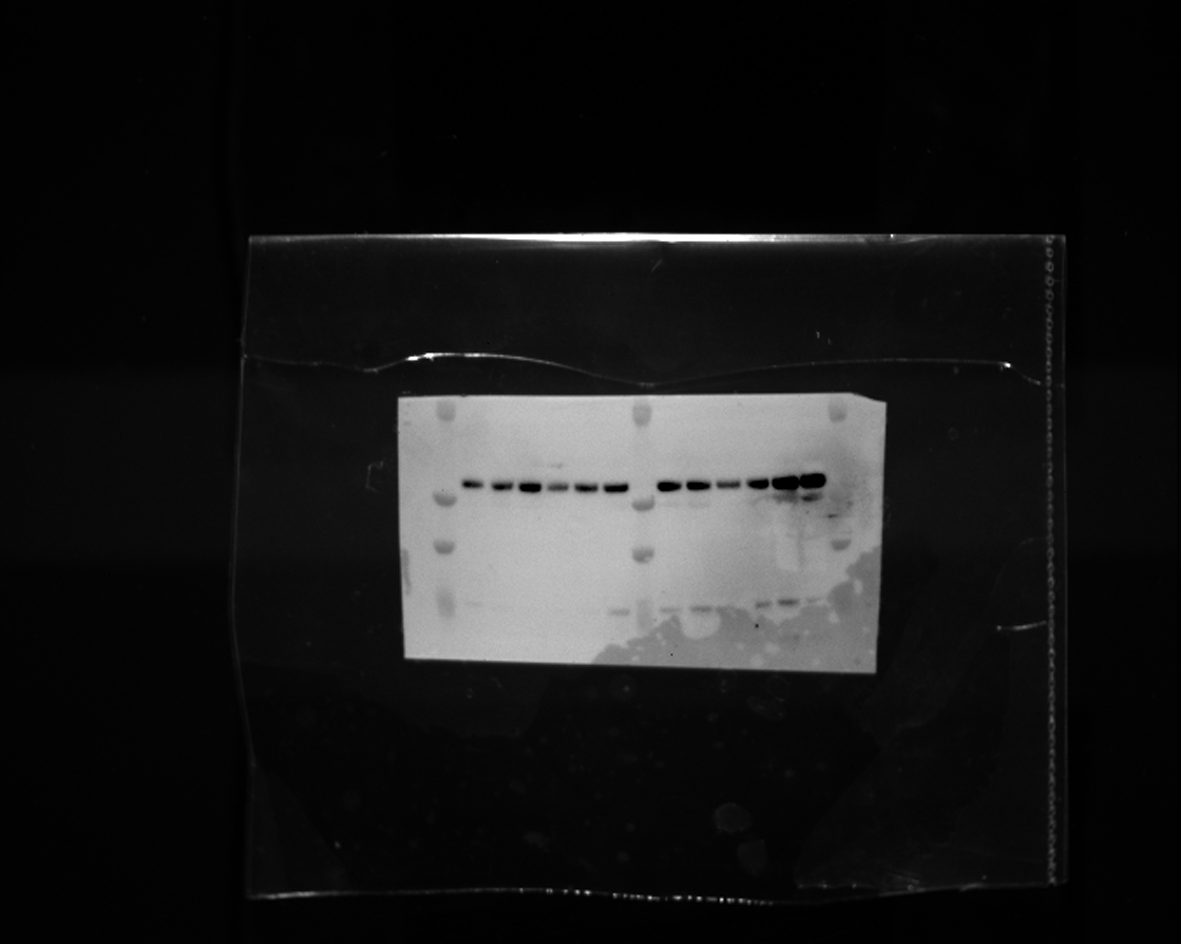

Supplement: Supplementary file 5 — Source data Fig. 4 [file 44321_2026_400_MOESM5_ESM.zip › Fig 4/Fig 4g/CHEMI_06042025_125725_(Chemi) occludin.tif]

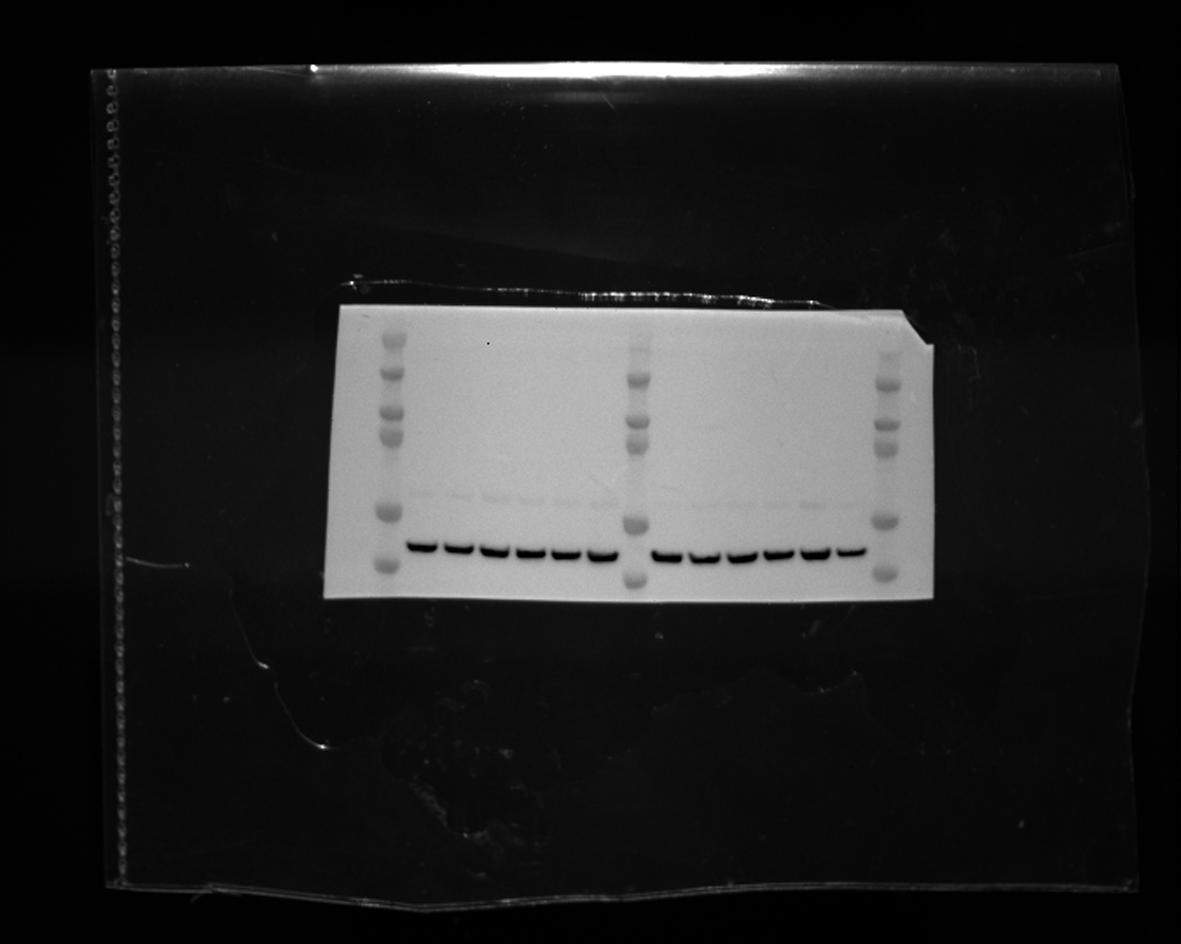

Supplement: Supplementary file 5 — Source data Fig. 4 [file 44321_2026_400_MOESM5_ESM.zip › Fig 4/Fig 4g/CHEMI_06092025_133243_(Chemi) actin for occludin.tif]

## Slide 1
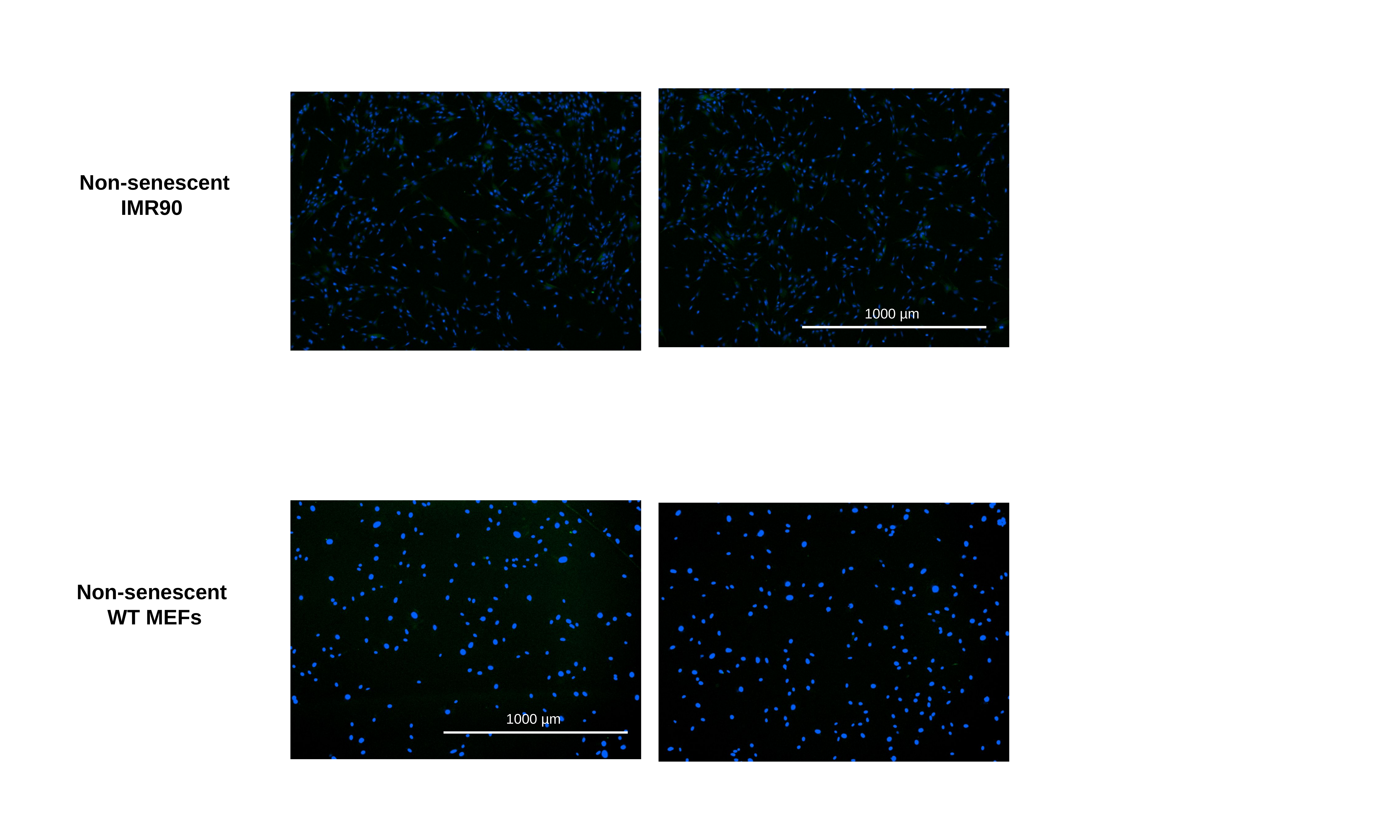

Non-senescent IMR90
1000 µm
Non-senescent
WT MEFs
1000 µm

Supplement: Supplementary file 6 — Figure EV1 Source Data [file 44321_2026_400_MOESM6_ESM.zip › Ext data Fig 1/Fig 1C Representative images - NS.pptx]

## Slide 1
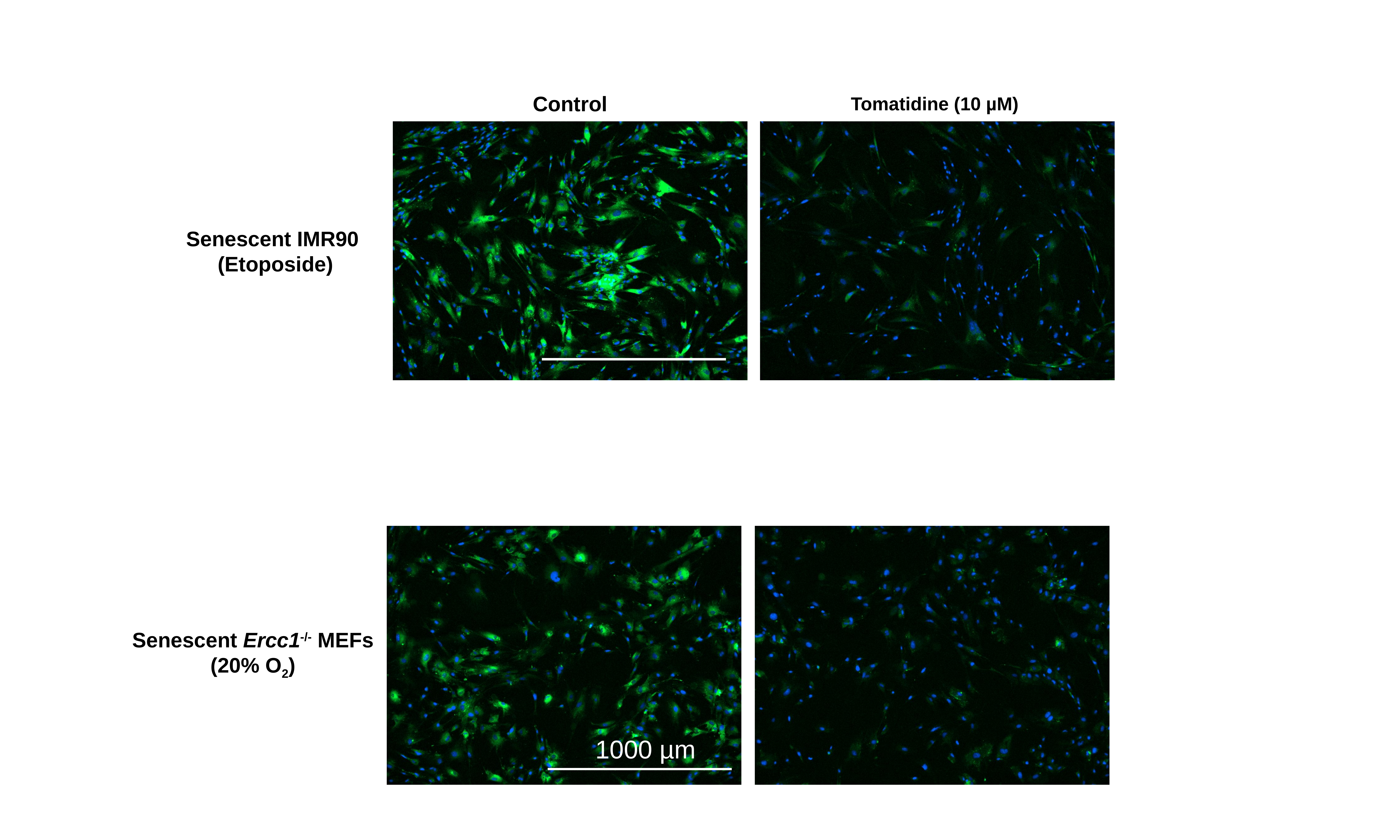

Control
Tomatidine (10 µM)
Senescent IMR90
(Etoposide)
Senescent Ercc1-/- MEFs
(20% O2)
1000 µm

Supplement: Supplementary file 6 — Figure EV1 Source Data [file 44321_2026_400_MOESM6_ESM.zip › Ext data Fig 1/Fig 1C Representative images.pptx]

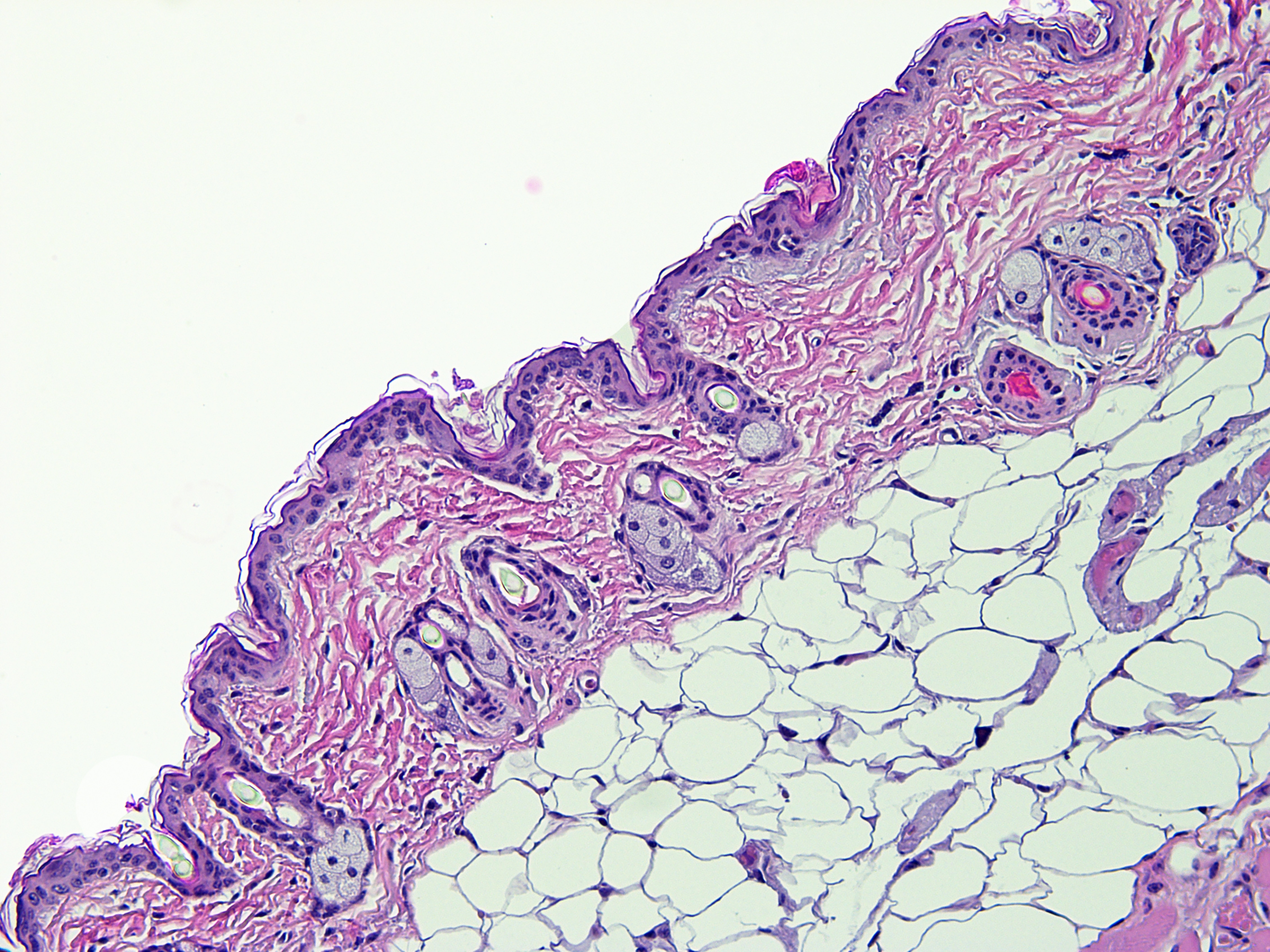

Supplement: Supplementary file 8 — Figure EV3 Source Data [file 44321_2026_400_MOESM8_ESM.zip › Ext data Fig 3/Tomatidine_DJ896_Series007_SV-1.jpg]

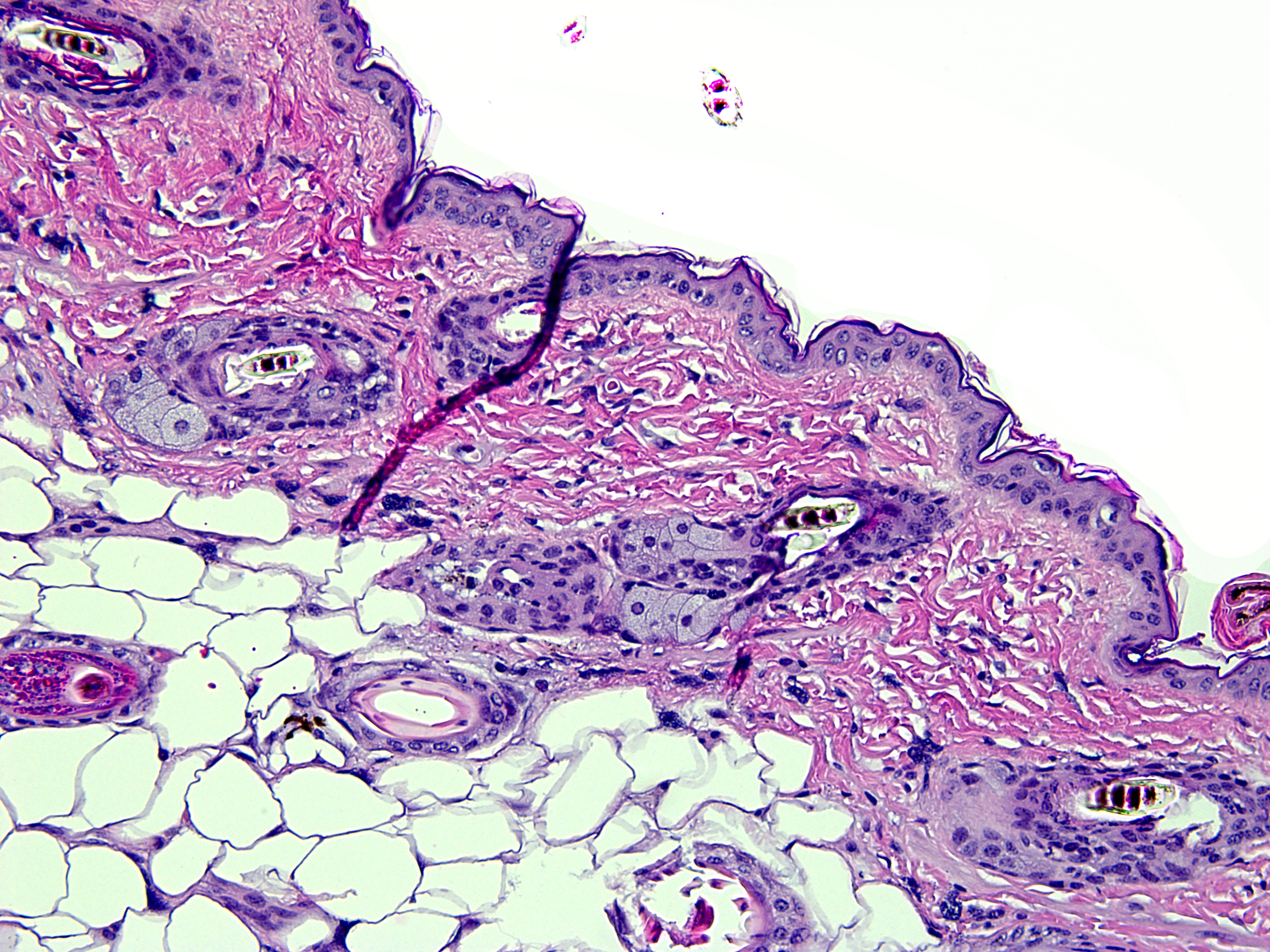

Supplement: Supplementary file 8 — Figure EV3 Source Data [file 44321_2026_400_MOESM8_ESM.zip › Ext data Fig 3/Vehicle_DJ892_Series004_SV.jpg]

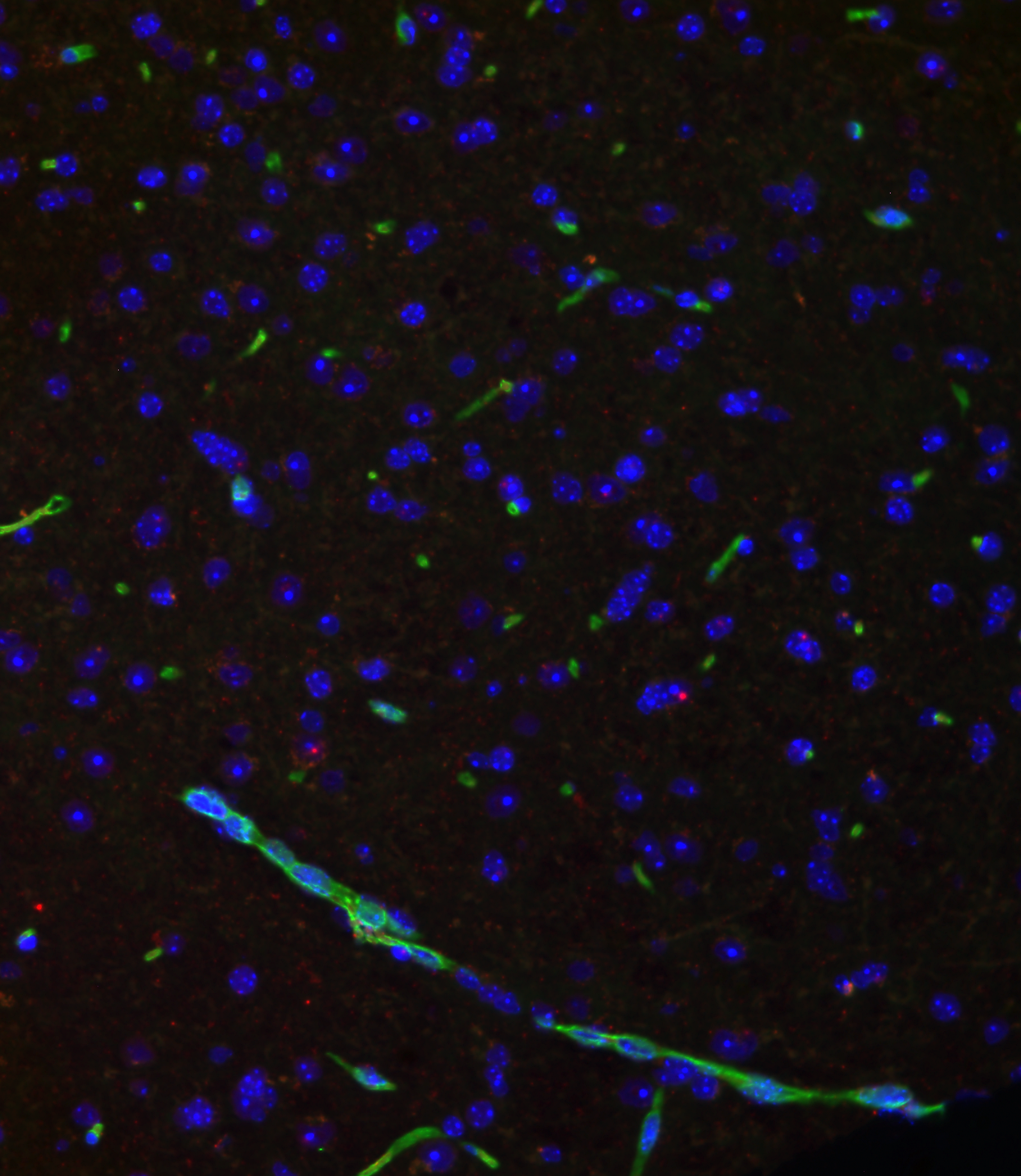

Supplement: Supplementary file 10 — Figure EV5 Source Data [file 44321_2026_400_MOESM10_ESM.zip › Ext data Fig 5/5a/BBB_DJ 875_Tomatidine_p21+Glut1_DGC.lif - Image 2.png]

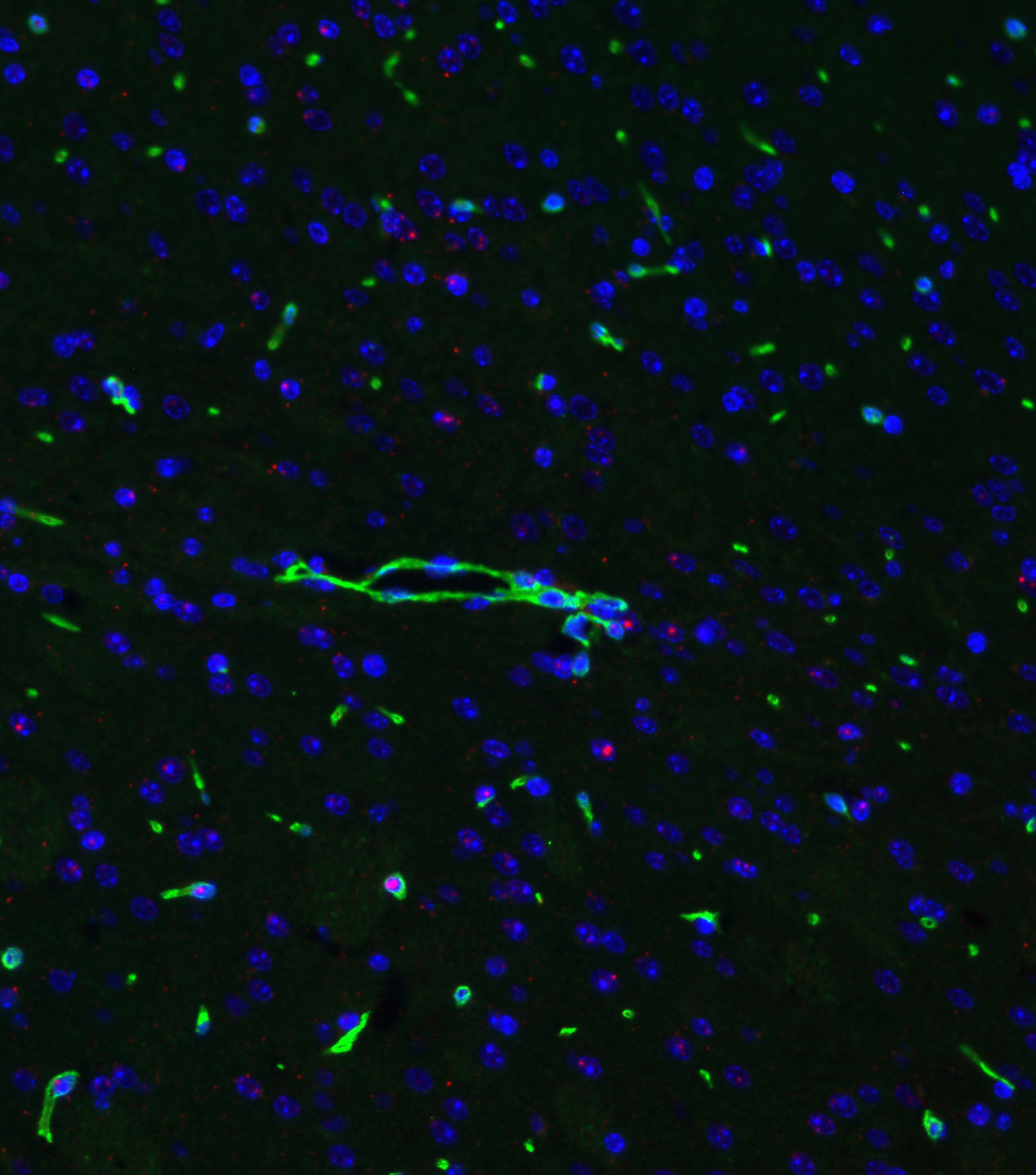

Supplement: Supplementary file 10 — Figure EV5 Source Data [file 44321_2026_400_MOESM10_ESM.zip › Ext data Fig 5/5a/BBB_DJ 885_Tomatidine_p21+Glu1_DGC.lif - Image 5.png]

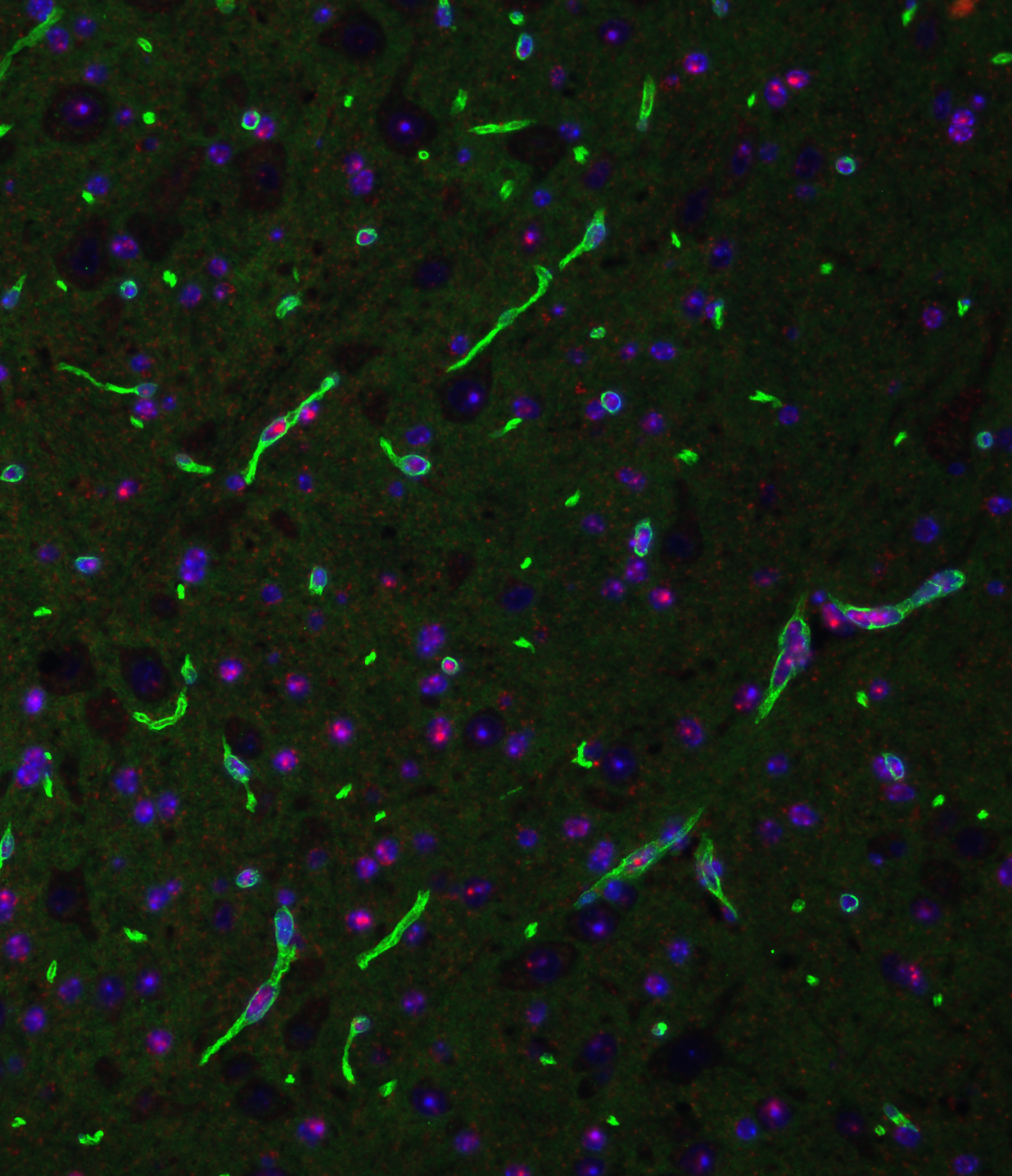

Supplement: Supplementary file 10 — Figure EV5 Source Data [file 44321_2026_400_MOESM10_ESM.zip › Ext data Fig 5/5a/BBB_DJ 932_vehicle_P21+GLUT1_1_26_2025.lif - Image 3_400um wide_length.png]

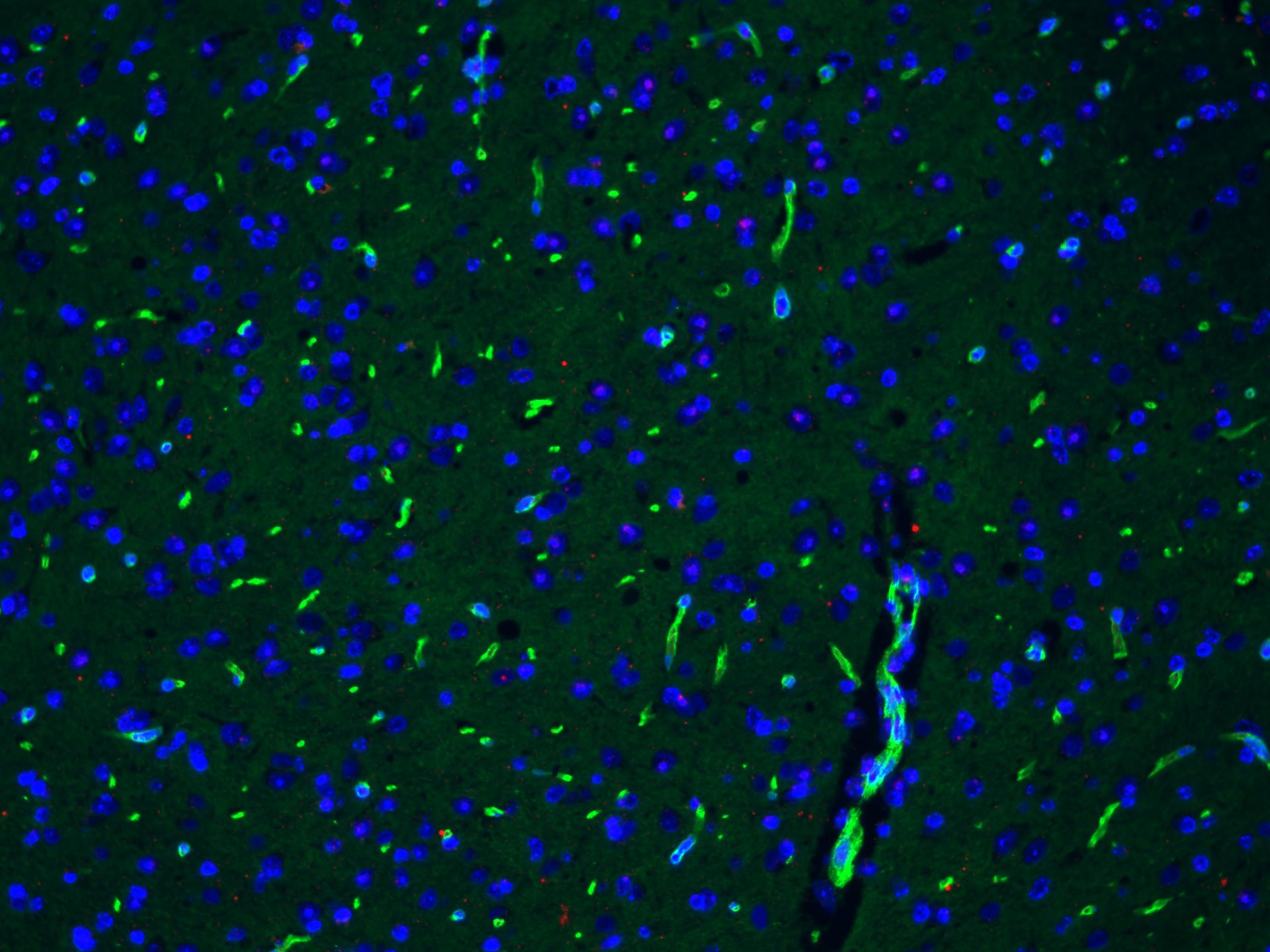

Supplement: Supplementary file 10 — Figure EV5 Source Data [file 44321_2026_400_MOESM10_ESM.zip › Ext data Fig 5/5b/BBB_DJ 881_Tomatidine_p16+Glut1_DGC.lif - Image 4.png]

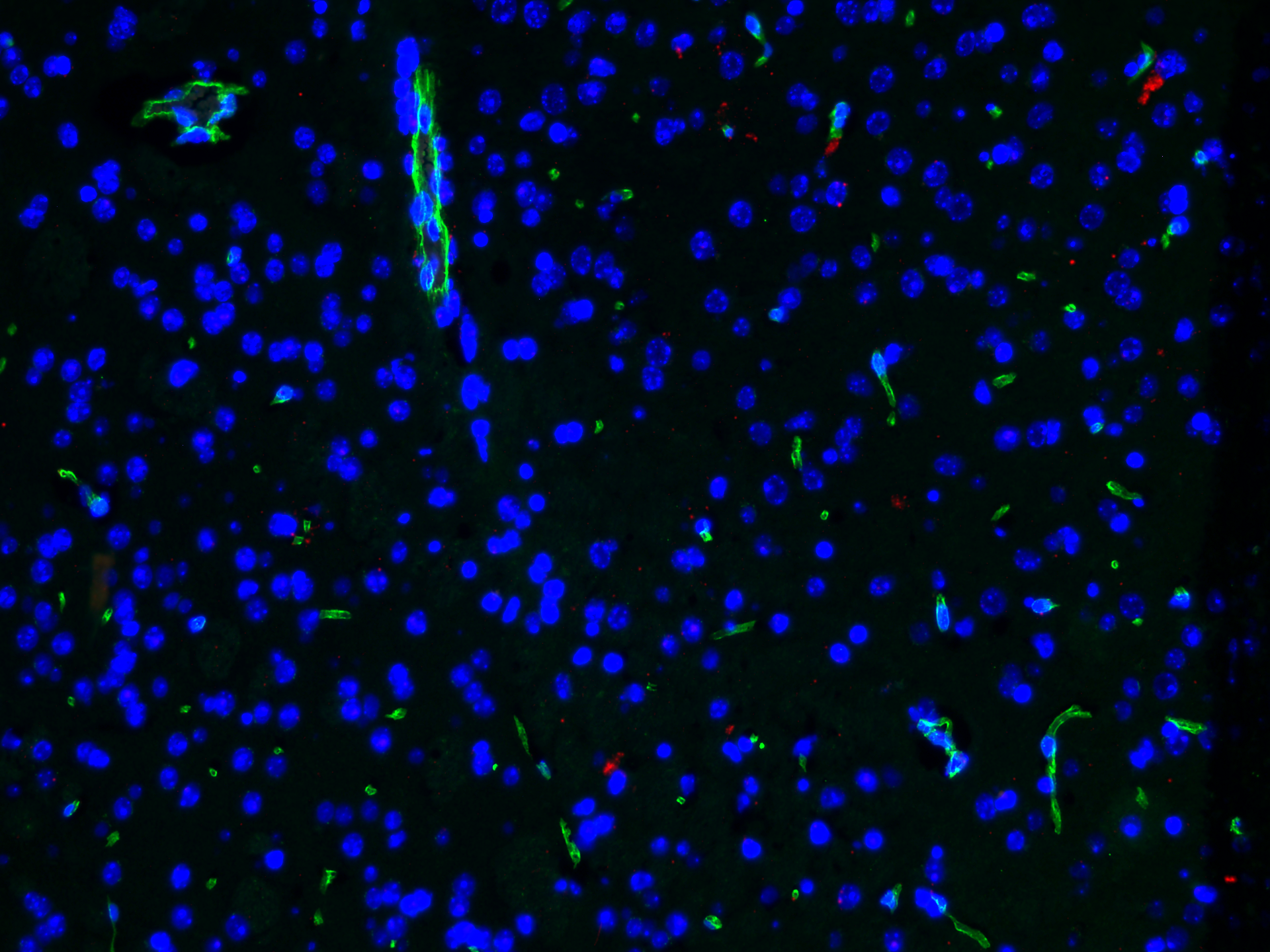

Supplement: Supplementary file 10 — Figure EV5 Source Data [file 44321_2026_400_MOESM10_ESM.zip › Ext data Fig 5/5b/BBB_DJ 896_Tomatidine_P16+Glut1_DGC.lif - Image 3.png]

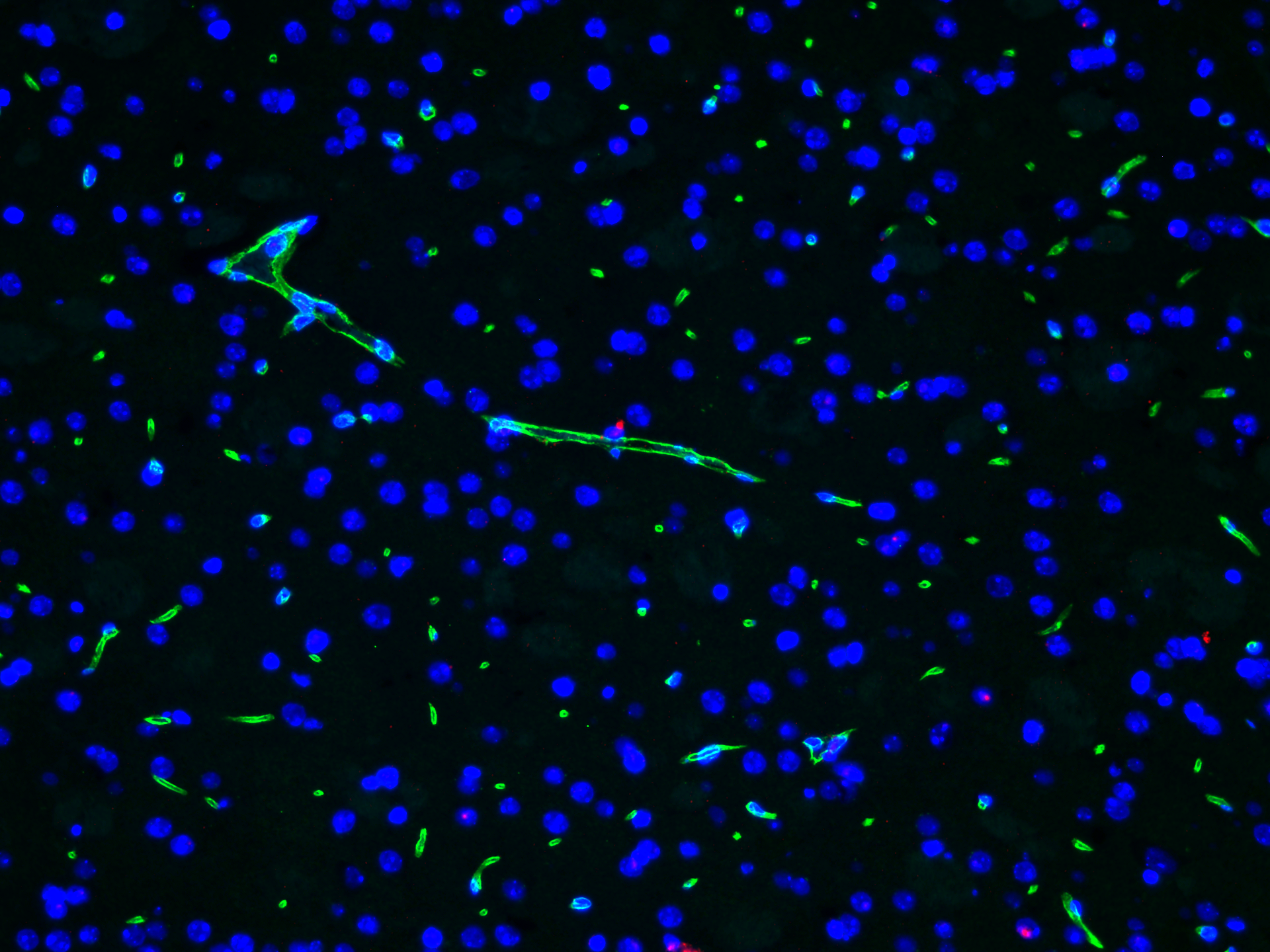

Supplement: Supplementary file 10 — Figure EV5 Source Data [file 44321_2026_400_MOESM10_ESM.zip › Ext data Fig 5/5b/BBB_DJ 934_Tomatidine_p16+Glut1_DGC.lif - Image 5.png]

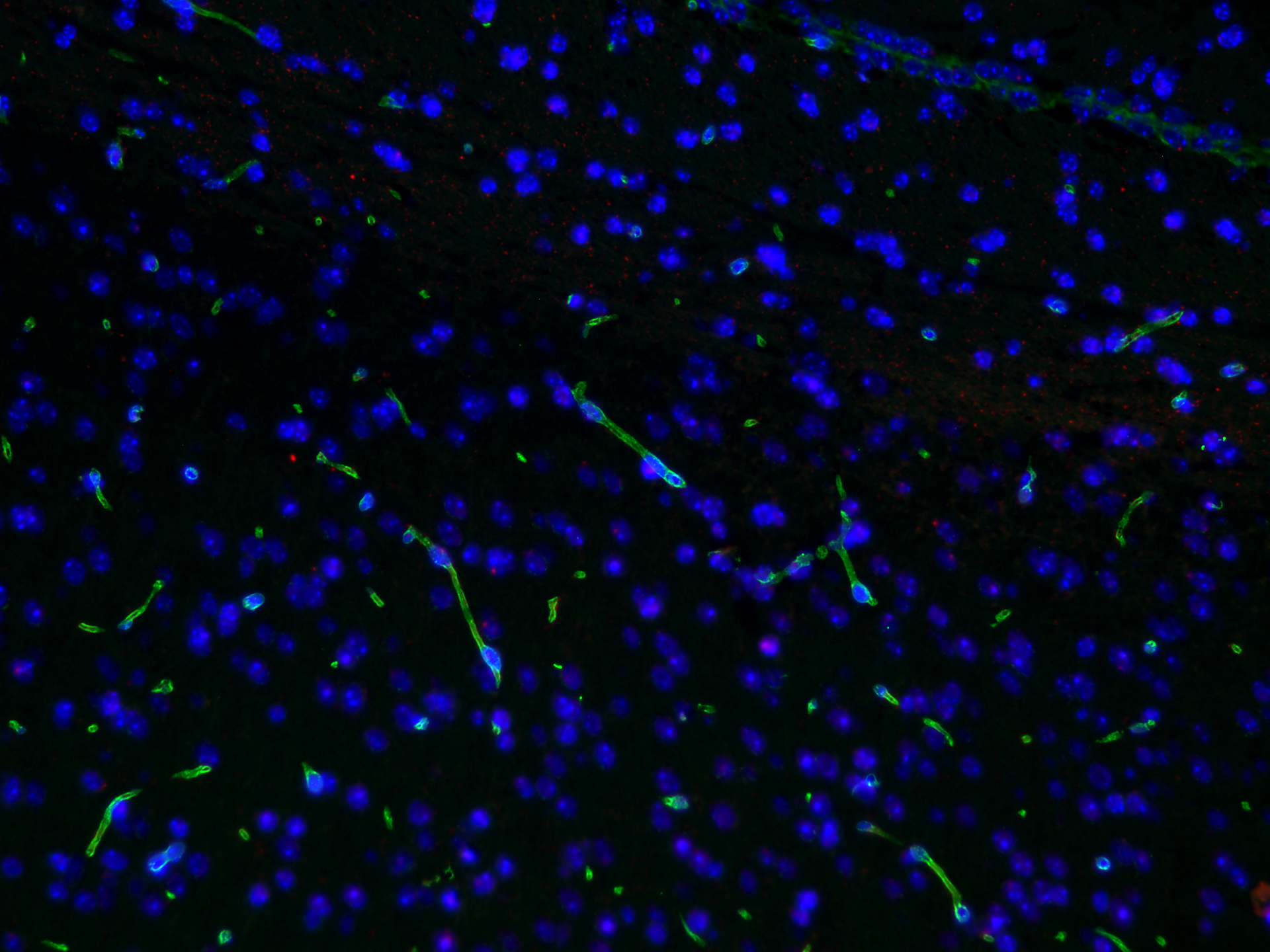

Supplement: Supplementary file 10 — Figure EV5 Source Data [file 44321_2026_400_MOESM10_ESM.zip › Ext data Fig 5/5b/BBB_DJ 938_tomatidine_p16+Glut1_DGC_vehicle_lif - Image 1.png]
