# Supplementary material for: Tomatidine is a senotherapeutic compound that improves cognitive function and reduces cellular senescence in aged mice
Source: EMBO Mol Med. 2026 Apr 1;18(5):1530–50. doi: 10.1038/s44321-026-00400-0 (PMC13179369; doi:10.1038/s44321-026-00400-0)
Supplement: Supplementary file 11 — Expanded View Figures [file 44321_2026_400_MOESM11_ESM.pdf]

Expanded View Figures

Figure EV1. Identification of tomatidine as a novel senomorphic compound.

(**A**) Schematic of the senescent cell-based screening workflow. Senescent cells were generated, seeded into 96-well plates, treated with compounds, stained with C12FDG (SA-β-gal) and DAPI, imaged on a high-content system, and analyzed. (**B**) Concentration-response curves of tomatidine in senescent Ercc1$^{-/-}$ MEFs and in total Ercc1$^{-/-}$ MEF cultures. (**C**) Concentration-response curves comparing senescent Ercc1$^{-/-}$ MEFs to non-senescent WT MEFs. Responses were normalized to untreated controls and fit by nonlinear regression using a variable-slope model (GraphPad Prism). The IC_{50} for senescent Ercc1^{-}/$^{-}$ MEFs was 1.24 (95% CI: 1.03–1.46), whereas the IC_{50} for non-senescent WT MEFs was 41.52 (95% CI: 21.42–140.5). The selectivity index (SI), calculated as IC_{50} (WT) / IC_{50} (senescent Ercc1^{-}/$^{-}$), was 33.5. Data were shown as mean ± SD from $n = 3$ independent experiments, technical replicates. "Total" denotes mixed cultures following the senescence-induction protocol. The non-senescent control data presented in panels (**B**, **C**) derive from the same experimental dataset. (**D**) Representative C12FDG fluorescence images (green: C12FDG/SA-β-gal; blue: Hoechst). Scale bar, 100 µm. (**E**) Illustration of the experimental setup for senomorphic assays. (**F**, **G**) Relative mRNA expression of p16, p21, and SASP-related genes in senescent IMR90 fibroblasts treated with tomatidine (in **F**) $p < 0.0001$ and in (**G**) IL6 $p = 0.021$ and $p = 0.005$, IL8 $p < 0.0001$ and $p < 0.0001$, IL1a $p = 0.001$ and $p = 0.031$, IL-1b < 0.0001 and $p < 0.0001$). (**H**, **I**) Relative mRNA expression of p16, p21, and SASP-related genes in senescent HBMECs treated with tomatidine. (for both p16 and p21 in **H**): $p < 0.0001$, in (**I**): IL-6 $p = 0.0006$ and $p < 0.0001$, IL1a: $p < 0.0001$ and $p < 0.0001$, IL-1b: $p = 0.083$ and $p = 0.0008$, CCL2: $p < 0.0001$ and $p < 0.0001$). (**J**) Illustration of the experimental setup for senolytic assays. (**K**) Relative mRNA expression of p16 ($p = 0.0102$) and p21 in senescent HBMECs after 48 h of tomatidine treatment. Data were presented as mean ± s.e.m. (**F–K**). Statistical significance was determined using two-way ANOVA followed by Tukey's multiple comparisons test. $n = 3$, technical replicates. Source data are available online for this figure.

© The Author(s)

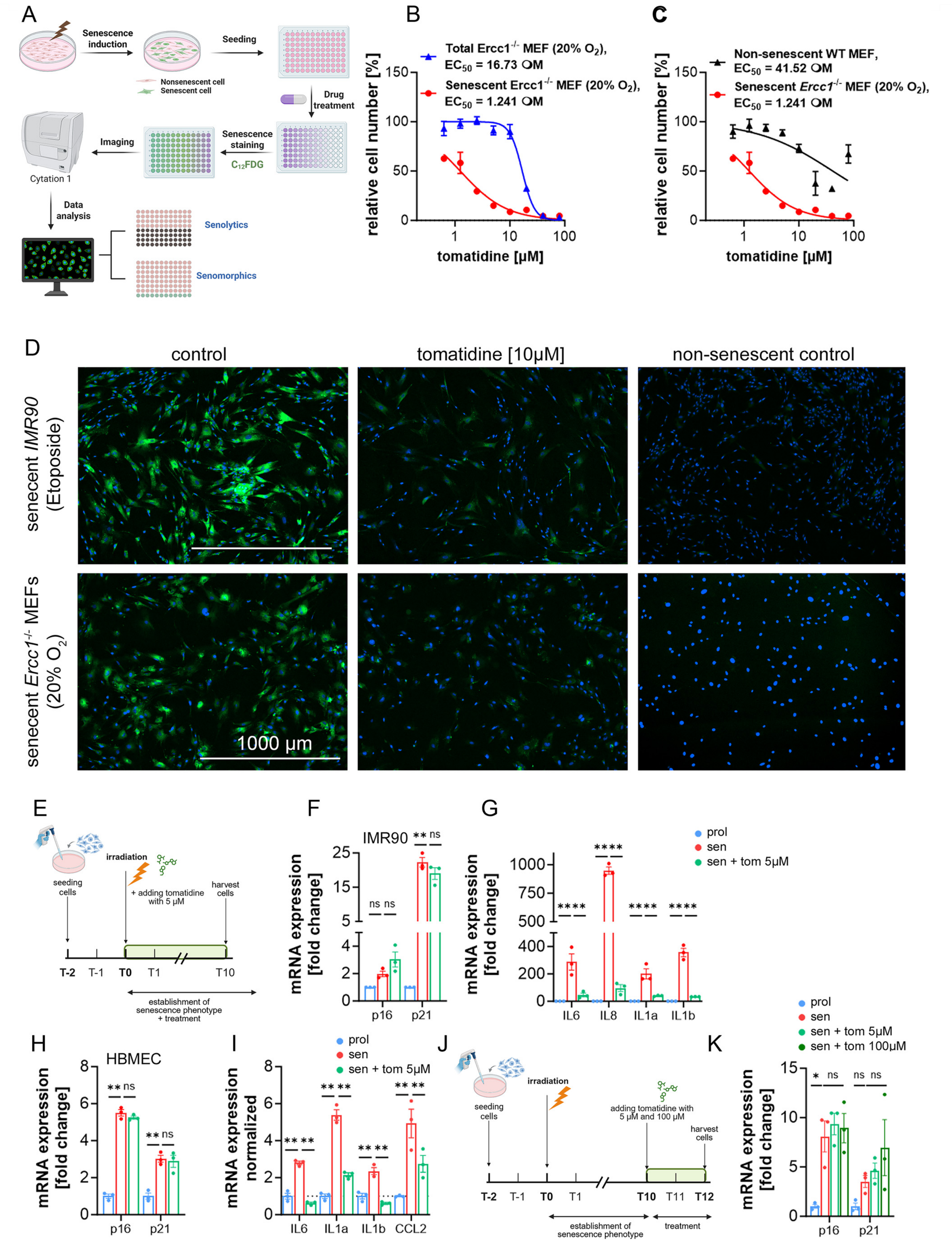

A
Senescence induction
Seeding
Nonsenescent cell
Senescent cell
Drug treatment
Senescence staining
C12FDG
Imaging
Cytation 1
Data analysis
Senolytics
Senomorphics
B
Total Ercc1-/- MEF (20% O2), EC50 = 16.73 µM
Senescent Ercc1-/- MEF (20% O2), EC50 = 1.241 µM
relative cell number [%]
tomatidine [µM]
C
Non-senescent WT MEF, EC50 = 41.52 µM
Senescent Ercc1-/- MEF (20% O2), EC50 = 1.241 µM
D
control
tomatidine [10µM]
non-senescent control
senecent IMR90 (Etoposide)
senecent Ercc1-/- MEFs (20% O2)
1000 µm
E
seeding cells
irradiation
+ adding tomatidine with 5 µM
harvest cells
T-2 T-1 T0 T1 T10
establishment of senescence phenotype + treatment
F
IMR90
mRNA expression [fold change]
p16 p21
G
prol
sen
sen + tom 5µM
IL6 IL8 IL1a IL1b
H
HBMEC
I
mRNA expression normalized
IL6 IL1a IL1b CCL2
J
adding tomatidine with 5 µM and 100 µM
T-2 T-1 T0 T1 T10 T11 T12
establishment of senescence phenotype
treatment
K
sen + tom 100µM

 © The Author(s)

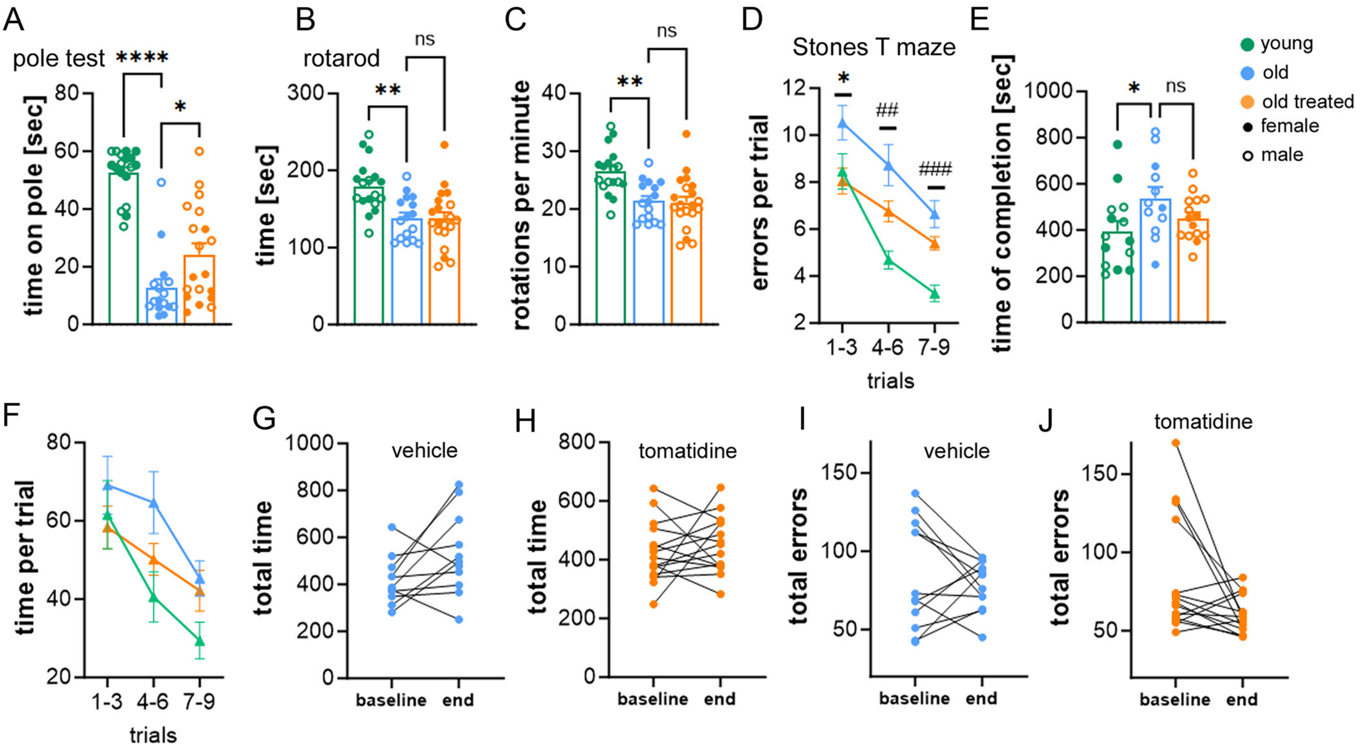

Figure EV2. Healthspan and cognitive assessments in aged mice treated with tomatidine.

(**A**) Performance on the pole test, measuring motor coordination and balance. (**B**, **C**) Rotarod testing showing (**B**) time spent on the rod and (**C**) maximal speed achieved. (**D**) Number of errors made in the Stone T maze across trials. Symbols: (*) indicates a significant difference between old and old tomatidine-treated groups, (##) indicates significant differences between young and old groups. Significant improvements in errors made between trial 1–3 vs 7–9 ($p = 0.0003$) and 4–6 vs 7–9 ($p = 0.0092$) in treated animals and in untreated animals, significant improvements between trial 1–3 vs 7–9 ($p = 0.0014$). (**E**, **F**) Time required to complete the Stone T maze in aged control and aged tomatidine-treated mice. (**G**, **H**) Comparison of baseline versus endpoint measurements for total time spent completing the maze. (**I**, **J**) Comparison of baseline versus endpoint measurements for total errors made prior to completing the maze. Data were presented as mean ± s.e.m. Statistical significance was determined using one-way or two-way ANOVA followed by Tukey's multiple comparisons test. $n = 15$–23, biological replicates. Males are represented by open circles, females by filled circles. Source data are available online for this figure.

© The Author(s)

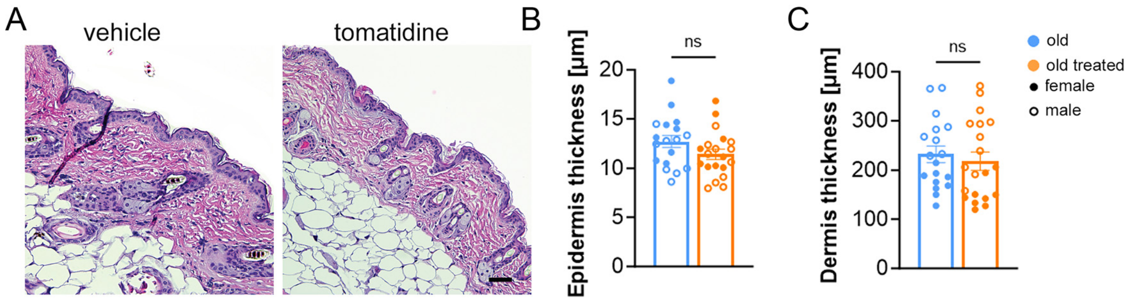

Figure EV3. Skin thickness measurements in aged mice treated with tomatidine.

(**A**) Representative H&E-stained skin sections from tomatidine-treated aged mice and untreated aged controls. (**B**) Quantification of epidermis thickness ($p = 0.1238$). (**C**) Quantification of dermal thickness ($p = 0.5839$). Data were presented as mean ± s.e.m. Statistical significance was determined using a *t*-test. Scale bar = 50 µm, $n = 18$–21, biological replicates. Males are represented by open circles, females by filled circles. Source data are available online for this figure.

 © The Author(s)

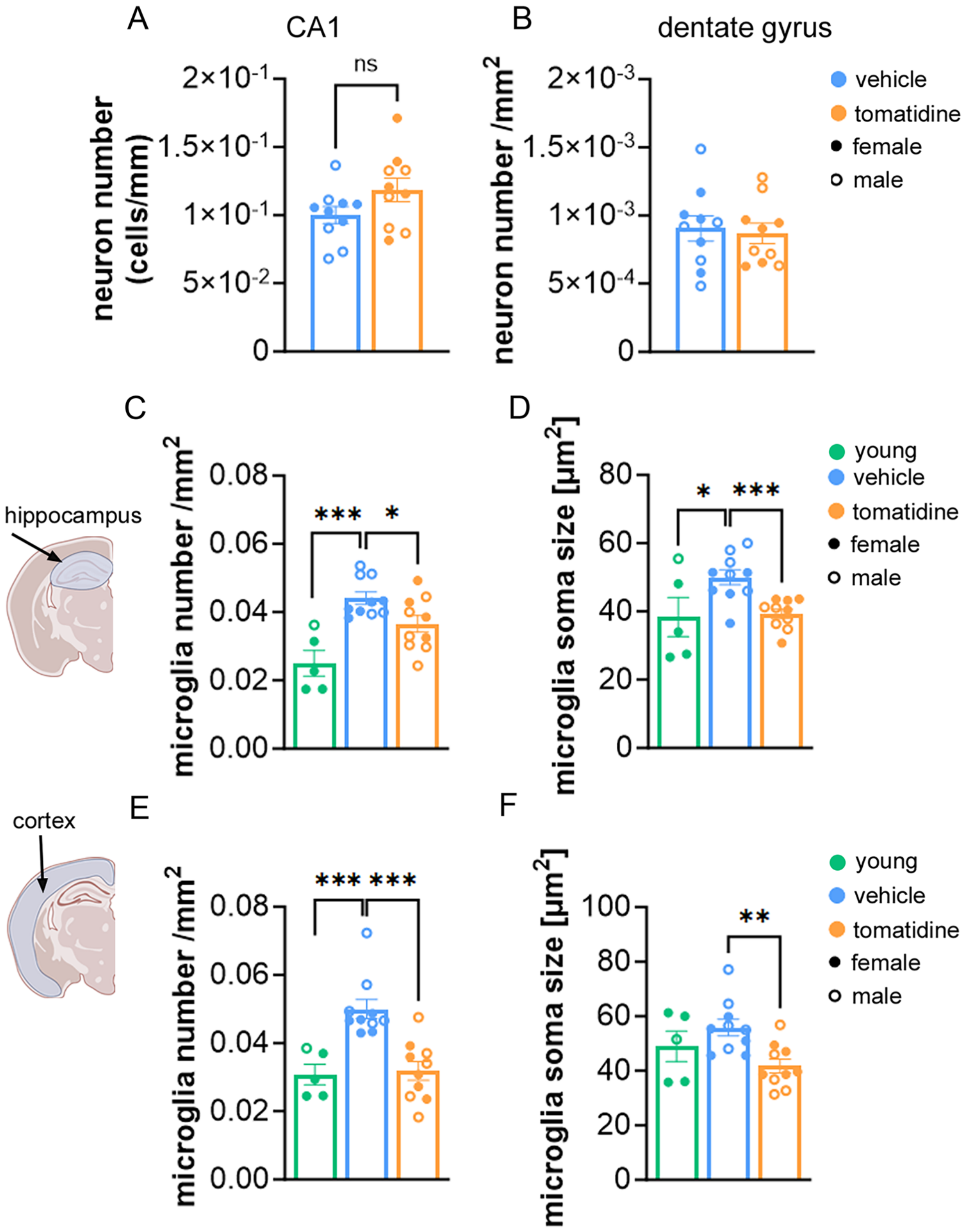

Figure EV4. Neuronal density and microglial morphology in hippocampus and cortex of aged mice treated with tomatidine.

(**A**) Quantification of neuronal density in CA1, showing the number of NeuN⁺ neurons per mm length ($p = 0.0501$). (**B**) Neuronal density in the dentate gyrus (DG) region of the hippocampus ($p = 0.3802$). (**C**) Number of Iba1⁺ microglia in the hippocampus; Exact p values for number Iba1⁺ (Young vs. vehicle, $p = 0.0002$; vehicle vs. tomatidine, $p = 0.0230$). (**D**) Microglial soma size in the hippocampus, reflecting changes in activation state; Exact p values for soma size in hippocampus (young vs. vehicle, $p = 0.037$; vehicle vs. tomatidine, $p = 0.0003$). (**E**) Number of Iba1⁺ microglia in the cortex; Exact p values for number Iba1⁺ (Young vs. vehicle, $p = 0.0009$; vehicle vs. tomatidine, $p = 0.0002$). (**F**) Microglial soma size in the cortex; Exact p values for soma size in cortex (Young vs. vehicle, $p = 0.2525$; vehicle vs. tomatidine, $p = 0.0022$). Panels (**C–F**) present the same datasets included in the main figure; here, we additionally show the values from young control mice to allow direct comparison between young, aged, and tomatidine-treated aged groups. Data were presented as mean ± s.e.m. Statistical significance was determined using a t-test or one-way ANOVA. $n = 5$-10, biological replicates. Males are represented by open circles, females by filled circles. Source data are available online for this figure.

© The Author(s)

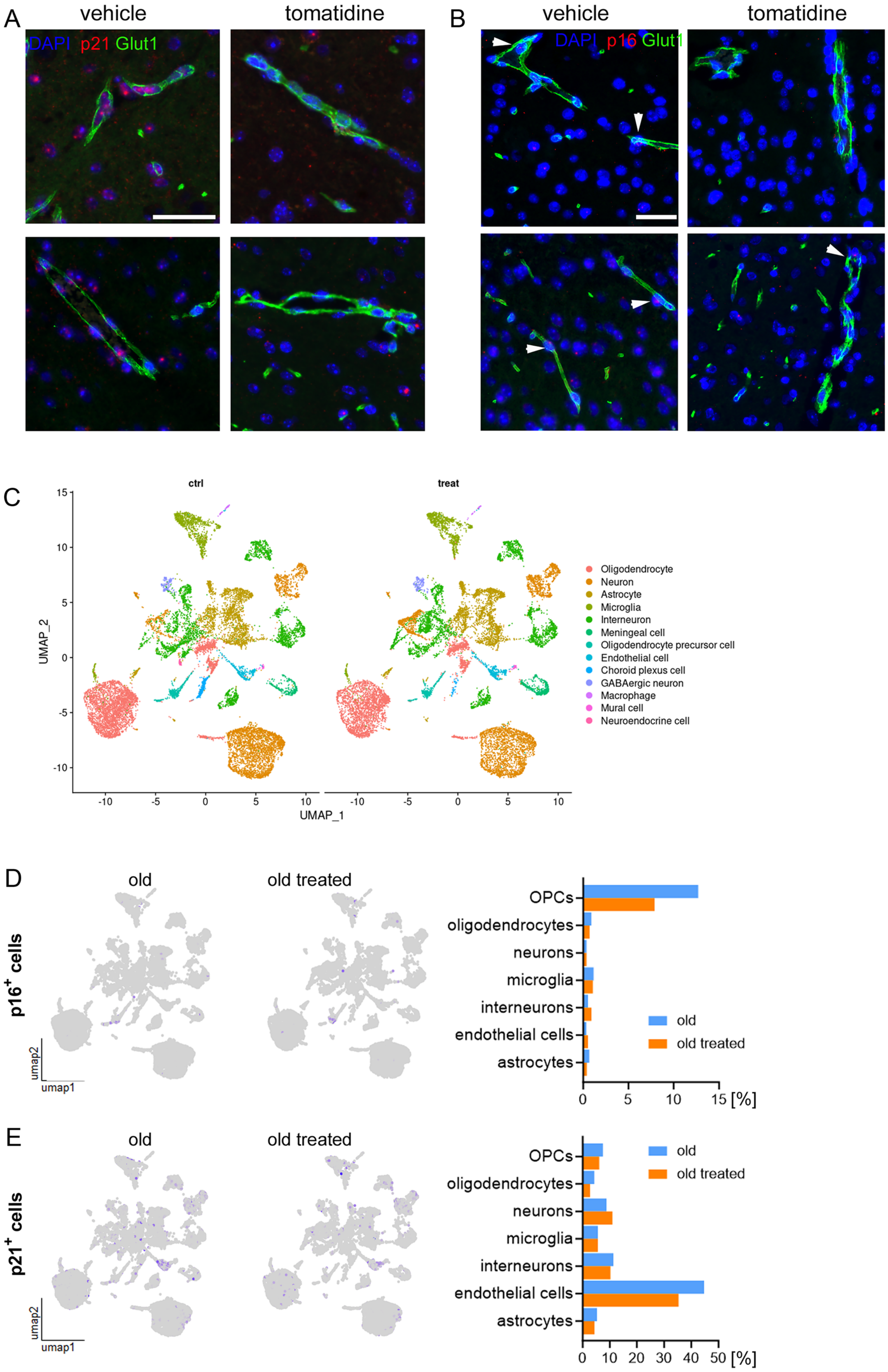
A
vehicle
tomatidine
DAPI p21 Glut1
B
vehicle
tomatidine
DAPI p16 Glut1
C
ctrl
treat
UMAP_2
UMAP_1
Oligodendrocyte
Neuron
Astrocyte
Microglia
Interneuron
Meningeal cell
Oligodendrocyte precursor cell
Endothelial cell
Choroid plexus cell
GABAergic neuron
Macrophage
Mural cell
Neuroendocrine cell
D
old
old treated
p16+ cells
umap2
umap1
OPCs
oligodendrocytes
neurons
microglia
interneurons
endothelial cells
astrocytes
old
old treated
0 5 10 15 [%]
E
old
old treated
p21+ cells
umap2
umap1
OPCs
oligodendrocytes
neurons
microglia
interneurons
endothelial cells
astrocytes
old
old treated
0 10 20 30 40 50 [%]

 © The Author(s)

Figure EV5. RNA-ISH and scRNA-seq analysis of p16 and p21 expression in brain endothelial cells.

(**A**) Representative RNA-ISH images showing p21 mRNA expression in GLUT1-positive brain endothelial cells (blue: DAPI; red: p21; green: GLUT1). Scale bar 50 μm. (**B**) Representative RNA-ISH images showing p16 mRNA expression in GLUT1 positive BBB endothelial cells (blue: DAPI; red: p16; green: GLUT1). White arrows indicate positive endothelial cells. Scale bar 30 μm (**C**) UMAP representation of all cell types colored by class from tomatidine treated and untreated controls. scRNA-seq was performed on hippocampus pooled from four animals per group. (**D**) UMAP representation and quantification of $p16^{+}$ cells from tomatidine treated and untreated controls. (**E**) UMAP representation and quantification of $p21^{+}$ cells from tomatidine treated and untreated controls. Source data are available online for this figure.

© The Author(s)
